# Metabolic reprogramming of CAR T cells: a new frontier in cancer immunotherapy

**DOI:** 10.3389/fimmu.2025.1688995

**Published:** 2025-11-19

**Authors:** Tjaša Frlic, Mojca Pavlin

**Affiliations:** Institute of Biophysics, Faculty of Medicine, University of Ljubljana, Ljubljana, Slovenia

**Keywords:** chimeric antigen receptor (CAR), adoptive cell immunotherapy, metabolic modulation, immunometabolism, persistence, T cell differentiation, exhaustion, epigenetics

## Abstract

Chimeric Antigen Receptor (CAR) T cell therapy has revolutionized hematological cancer treatment, but its efficacy in solid tumors remains limited by the immunosuppressive and metabolically hostile tumor microenvironment (TME). CAR T cells’ functional compromise, exhaustion, and poor persistence are critically linked to their suboptimal metabolic fitness. This review highlights a paradigm shift: immunometabolism and its intricate interplay with epigenetics profoundly regulate T cell fate and function, establishing their reprogramming as a cornerstone for optimizing CAR T cell efficacy in diverse malignancies. We explore the intricate relationship between T cell differentiation and metabolic states, emphasizing that modulating CAR T cell metabolism *ex vivo* during manufacturing can drive differentiation towards less exhausted, more persistent memory phenotypes, such as stem cell central memory (T_scm_) and central memory (T_cm_) cells, which correlate with superior anti-tumor responses. Our analysis demonstrates that metabolic inhibitors offer significant potential to reprogram CAR T cells. Agents targeting glycolysis or the PI3K/Akt/mTOR pathway promote a memory-like phenotype by favoring oxidative phosphorylation (OXPHOS). Further strategies utilizing glutamine antagonists, mitochondrial modulators, or enzyme manipulation (e.g., IDH2, ACAT1) can epigenetically reprogram cells, fostering memory and exhaustion resistance. Similarly, nutrient level optimization during *ex vivo* expansion directly sculpts CAR T cell metabolic profiles. With approaches like glucose restriction/galactose substitution, or specific amino acid modulation (e.g., L-arginine, asparagine), persistence of CAR T cells in patients can be improved. The judicious selection and engineering of cytokines (e.g., IL-7, IL-15, IL-21) during manufacturing also plays a vital role in fostering desired memory phenotypes. In conclusion, metabolic engineering, leveraging its impact on epigenetic regulation during CAR T cell manufacturing, is crucial for generating potent, persistent, and functionally resilient products. This approach holds immense promise for expanding the curative potential of CAR T cell therapy to a broader range of cancers, particularly challenging solid tumors.

## 1 Introduction

In the past decade, the development of immunotherapies has revolutionized cancer treatment, offering improved efficacy and reduced side effects compared to traditional treatments. Therapy with modified T cells expressing chimeric receptors for tumor antigens CAR T (chimeric antigen receptor CAR) is a novel and highly significant immunotherapeutic approach, already approved and widely utilized in clinical settings for hematological malignancies (1–6). A CAR is an engineered receptor expressed in T cells that comprises an extracellular antigen-binding domain, a transmembrane domain, and intracellular signaling domains. This enables specific recognition of antigens on tumor cells and subsequent initiation of an immune response. Genetically modified CAR T cells are designed to express extracellular antigen-binding domain, typically a single-chain variable fragment (scFv) derived from antibody variable regions, which specifically recognizes tumor antigens in a non-MHC-restricted manner (7). Membrane domains such as CD4, CD8, CD28, or CD3ξ anchor the receptor to the cell membrane, while the activation domain, commonly the CD3ζ signaling domain of the T cell receptor, initiates the T cell activation signal. Finally, costimulatory domains enhance T cell activation, cytokine secretion, proliferation and persistence (8–10).

In first-generation, chimeric receptors possessed singular intracellular domain, typically CD3ζ. The primary limitation of these constructs was the absence of a costimulatory domain, which resulted in the relatively short persistence of these cells in patients (7, 11, 12). Second-generation receptors were improved with an additional costimulatory domain (such as the CD28 or 4-1BB receptor) alongside CD3ζ, thereby enabling extended persistence of CAR T cells in patients (13, 14). Second-generation CAR T cells formed the basis for the first Federal Office for Food and Drug Administration (FDA) approved therapy in 2017, and all commercially available CAR T cell products currently utilize this type of construct (15). CAR T cell efficacy can be further improved by incorporating two sequential costimulatory domains, which is typical for third-generation CAR T cells (16). While CD28 domain boosts effector T cell function, 4-1BB domains is crucial for T cell memory phenotype, enabling prolonged anti-tumor effects (17). Third-generation CAR T cells are presently under clinical investigation for their potential to improve efficacy and minimize side effects in various cancer types (18–24). In addition, fourth-generation receptors, also known as TRUCKs (T cells Redirected for Universal Cytokine Killing), were designed to modify the TME and recruit other immune cells to generate a robust immune response by secreting cytokines (e.g. IL-12 and IL-15) into the tumor (25–27). Clinical trials involving fourth and next-generation CAR T cell therapy are ongoing, however, their precise clinical characteristic are largely unknown, necessitating further efforts for successful clinical translation (28–32).

Cell therapies necessitate a complex final product with stringent monitoring of critical process parameters and quality control to ensure safety and effectiveness. This complexity is further compounded by significant process variability, particularly arising from donor-specific differences. Consequently, producing cells for therapeutic purposes remains challenging (33), and understanding the impact of process parameters on T cells, in particular, remains limited. The choice of reagents, including media, cytokines, and supplements, as well as the duration of expansion, can have a substantial impact on the potency of *ex vivo* CAR T cells (34). At present, there is significant variation in the protocols used to produce CAR T cells (34), and standardization in this field has yet to be achieved. The general manufacturing procedure for CAR T cells typically begins with the isolation of peripheral blood mononuclear cells (PBMCs) containing T cells with leukapheresis. To achieve the purest collection of CD4^+^ and CD8^+^ T lymphocytes, cells are enriched and subsequently activated to promote T cell proliferation and differentiation. Next, T cells are transduced, most commonly using lentiviral vectors or gamma-retroviral vectors encoding CAR (35, 36). Finally, CAR T cells are expanded *ex vivo*, cryopreserved and re-infused intravenously into the patient (37). Before CAR T cell injection, patients commonly receive lymphodepleting chemotherapy to enhance CAR T cell persistence and proliferation (38).

To date, FDA has approved seven CAR T cell therapies (**Table 1**). The first five therapies target the CD19 antigen, prevalent in B-cell malignancies, while the latter two target BCMA (B maturation antigen), characteristic of multiple myeloma patients. These treatments have achieved remarkable success, with overall response rate between 30-70% and, in some trials, exceeding 90% (39–44, 46, 47).

**Table 1:** CAR T cell therapies approved by the FDA.

Antigen	CAR T product	Manufacturer	Indications	Ref.
CD19	Tisagenlecleucel	Novartis	R/R B-ALL, R/R DLBCL, R/R FL	(39–41)
	Axicabtagene ciloleucel	Gilead-Kite/FOSUN Kite	R/R DLBCL, transformed FL, PMBCL, and HGBCL	(42)
	Brexucabtagene autoleucel	Gilead-Kite	R/R MCL	(43)
	Lisocabtagene maraleucel	BMS-Juno	R/R DLBCL, HGBCL, PMBCL, FL grade 3B	(44)
	Obecabtagene autoleucel	Autolus Therapeutics plc	R/R B-ALL	(45)
BCMA	Idecabtagene vicleucel	BMS-Bluebird	R/R MM	(46)
	Ciltacabtagene autoleucel	Legend Biotech	R/R MM	(47)

R/R, relapse/refractory; B-ALL, B-cell acute lymphocytic leukemia; NHL, non-Hodgkin lymphoma; MM, multiple myeloma; DLBCL, Diffuse Large B-Cell Lymphoma; HGBCL, High-grade B-cell lymphoma; FL, Follicular lymphoma; PMBCL, Primary mediastinal large B cell lymphoma; MCL, Mantle cell lymphoma.

Regrettably, many studies continue to report CAR T cell ineffectiveness due to poor persistence in the patient (48–50) and an inability to achieve complete remission in certain disease types (51–53). It has become unequivocally clear that long-term persistence of CAR T cells is paramount for achieving complete and durable responses. CAR T cell persistence is largely influenced by factors, such as T cell phenotype (e.g. subset composition, differentiation, exhaustion), manufacturing and design factors (e.g. CAR construct, cytokine usage during expansion, time of expansion) and patient-specific factors. Therefore, significant efforts have been made towards increasing the *in vivo* persistence of CAR T cell therapy products.

In this review, we discuss the latest research on improving the characteristics and longevity of CAR T cells by modulating their metabolism. This involves the addition of metabolic modulators that target specific metabolic pathways or the alteration of nutrient levels during *ex vivo* preparation of CAR T cell product. These approaches could be readily incorporated into current CAR T cell manufacturing process without significant alterations to existing protocols. This metabolic enhancement strategy holds considerable promise for overcoming some of the current limitations of CAR T cell therapy, potentially leading to more durable and effective treatments for cancer patients.

## 2 Metabolic modulation of CAR T cells

In the following section, we will briefly overview the basic T cell phenotypes with associated metabolic alterations, the metabolic alterations during T cell activation, and their importance for the generation of persistent and effective CAR T cells.

### 2.1 Metabolic pathways shaping T cell differentiation and CAR T cell efficacy

CAR T cell persistence and therapeutic efficacy are intricately linked to their differentiation status and metabolic pathways. Critically, the modulation of metabolic pathways can directly influence T cell differentiation and function. Many pathways, transcription factors, and epigenetic modifications that affect T cell differentiation and persistence are closely linked to cellular metabolism (54–60).

T cells comprise various subsets, including naïve T cells (T_n_; CD45RA^+^, CCR7^+^, CD62L^+^, CD27^+^, CD95^-^), stem cell central memory T cells (T_scm_; CD45RA^+^, CCR7^+^, CD27^+^, CD62L^+^, CD95^+^), central memory T cells (T_cm_; CD45RA^-^CCR7^+^, CD27^+^,CD62L^+^, CD95^+^), effector memory T cells (T_em_; CD45RA^-^, CCR7^-^, CD27^-^, CD62L^-^, CD95^+^), effector T cells (T_eff_; CD45RA^-^, CCR7^-^, CD62L^-^, CD27^-^, CD95^+^), and terminally differentiated effector memory T cells (T_emra_; CD45RA^+^, CCR7^-^, CD62L^-^, CD27^-^, CD95^+^). The transition between these subtypes requires appropriate metabolic and epigenetic changes (61). Immature T lymphocytes are formed in the bone marrow and migrate to the thymus, where they acquire their T cell receptor (TCR) and mature into immunocompetent helper T cells (CD4^+^CD3^+^) or cytotoxic T lymphocytes (CD8^+^CD3^+^) (62).

T_n_ cells possess unique TCRs and constantly circulate, sampling antigens on major histocompatibility complexes (MHCs) (63). Once in the periphery, T_n_ cells exhibit low energy demands, primarily relying on oxidative phosphorylation (OXPHOS) or fatty acid oxidation (FAO) (64). Growth factors influence resting T_n_ cell longevity by regulating nutrient transporter expression (65), whereas autophagy can supply substrates for OXPHOS (66). Their quiescence is transcriptionally controlled by factors like forkhead box class O (FOXO), which also modulates metabolic functions and promotes lymphoid tissue homing, inducing the expression of CD62L, CC-chemokine receptor 7 (CCR7) and S1P receptor 1 (S1PR1) (67–69). S1P- induced signaling suppresses mitophagy in T_n_, preserving mitochondrial content and OXPHOS (70). Importantly, IL-7 signaling ensures the survival of T_n_ by increasing amino acid catabolism (65). Basal glycolytic flux is sustained by GLUT1, activated by IL-7R–AKT signals, with GLUT3 potentially contributing (71, 72).

The CAR T cell production process begins with obtaining T cells from the patient, which are mostly in a naïve state. Even without antigen exposure, certain CAR constructs can reprogram T_n_ cell metabolism, increasing glycolysis and nutrient uptake. This metabolic shift can compromise the naïve phenotype, leading to premature differentiation or exhaustion if not carefully controlled (73). The inhibition of FOXO1 in CAR T cells, furthermore resulted in a more exhausted phenotype and weakened anti-tumor response, whereas overexpression of FOXO1 enhanced anti-tumor immunity, increased mitochondrial mass, and induced stemness (74, 75).

During manufacturing process, T cells undergo activation, leading to a rapid increase in short-lived T_eff_ cells, while a small fraction differentiates into memory cells that can persist for years (76, 77). Long-lived memory cells are crucial for the efficacy of CAR T therapy (78). In contrast, when CAR T cells are exposed to target antigens, they must be able to activate, clonally expand, and mount efficient anti-tumor response (79). Below, we discuss the metabolic alterations of activated T cells and their importance in effective CAR T therapy.

### 2.2 Activation of CAR T cells and metabolic changes

During activation, T cells undergo a significant metabolic shift toward aerobic glycolysis, also known as the Warburg effect. This process rapidly generates ATP and provides intermediates for the biosynthesis of macromolecules, supporting clonal expansion and effector functions, such as cytokine secretion (61, 80–85).

TCR and costimulatory receptor signaling during activation trigger various pathways that regulate energy metabolism, with PI3K-Akt-mTOR being paramount. Cytokines like IL-2, together with CD28 ligation, activate and expand T cells by inducing PI3K-dependent Akt activation (83, 86, 87). This, in turn, stimulates the mammalian target of rapamycin (mTOR) kinase, which acts as a major nutrient level sensor and switch for anabolic metabolism, boosting glucose and amino acid consumption (83). mTORC1 complex alters anabolic and mitochondrial metabolism through transcriptional, translational and post- translational mechanisms: proteins synthesis is stimulated by phosphorylating 4E-BP1 and p70S6 kinase, and the synthesis of lipids through SREBP2 (88, 89). In contrast, mTORC2 contributes to quiescence exit, in part by repressing FOXO1 through Akt activation, which induces GLUT1 expression (88, 90). Both Akt as well as mTORC1 strongly stimulate aerobic glycolysis, enabling proliferation, differentiation, and effector functions. Transcription factors like Myelocytomatosis oncogene (c-Myc) and hypoxia-inducible factor 1 (HIF-1) also increase to enhance metabolic intermediates and T cell effector function; c-Myc enhances glycolysis and glutaminolysis enzymes, while HIF-1 boosts glucose uptake and glycolysis, enhancing T cell effector functions (80, 91).

Glycolysis rapidly generates ATP and provides critical intermediates for macromolecule biosynthesis, crucial for clonal expansion and effector functions (61, 80, 83, 92, 93). Another metabolic network required for T_eff_ cell function is upregulated mitochondrial fission, leading to a less efficient electron transport chain but enabling calcium buffering and production of reactive oxygen species (ROS) to support T cell activation (94, 95). ROS can activate the nuclear factor of activated T cells (NFAT), leading to the synthesis of IL-2 (96). Inhibiting fission promotes memory phenotype differentiation, decreasing inflammatory potential (97).

Efficient activation of CAR T cells plays a significant role in adequate tumor cell killing and overall therapeutic efficacy. The strength and duration of T cell activation can be modulated by various factors, including the design of the CAR or the metabolic state. The metabolic state before and during activation determines whether CAR T cells become potent effectors or exhausted cells (98). Specifically, a high glycolytic flux drives T cells toward a terminally differentiated state, whereas lower glycolysis favors memory T cell formation (99). Engineering and maintaining a metabolically favorable profile, especially one that balances glycolysis with mitochondrial health, is key to successful CAR T cell activation and long-term efficacy in patients.

The main purpose of the expansion triggered by activation and cytokines is to increase and enrich T cell numbers, and at the same time eliminate non-T cells by exposing them to culture conditions that are suboptimal to other cell types. Moderate and well-controlled *in vitro* proliferation is essential for generating potent CAR T cell products. The goal is not maximal expansion, but rather qualitative expansion that preserves the optimal cell subtypes (100). For example, transient cessation of CAR signaling before differentiation influences the developmental course of a substantial portion of CAR T cell populations, rather than fostering the proliferation of a small subset of highly proliferative cells (101). In addition, stronger tonic signaling directly impacts CAR T cell proliferation *in vitro*, consequently leading to CAR T cell exhaustion and diminished persistence and effectiveness *in vivo* (102).

### 2.3 The impact of co-stimulatory domains on CAR T cell metabolic profile and functionality

Following CAR T cell activation, lentiviral or retroviral gene transfer is conducted, which normally has no significant effect on cellular metabolism or mitochondrial dynamics. While gene transfer itself is metabolically neutral, the metabolic profile of T cell subsets can affect transduction efficiency and long-term survival. Furthermore, indirect effects may arise, such as tonic signaling from certain CAR constructs, as mentioned previously. Kawalekar et al. reported increased OXPHOS, mitochondrial spare respiratory capacity (SRC), and FAO in 4-1BB CAR T cells compared to CARs with CD28 (103). These differences arise from the molecular pathways triggered by each co-stimulatory domain. CD28 interacts with PI3K, leading to Akt activation, inducing glucose transporters and metabolic enzymes (104), while 4-1BB activates p38-MAPK signaling, resulting in PGC1α overexpression, mitochondrial fusion, and biogenesis (105, 106). Third-generation CAR T cells, with CD28 and 4-1BB co-stimulatory domains, maintain the high mitochondrial metabolism of 4-1BB CAR T cells, together with increased glycolytic metabolism. This enhanced metabolic activity drives sustained TCR signaling, proliferation, and metabolic fitness in dual CAR T cells (107). A more complete insight into the function of different domains applied in CAR T cell therapy and their influence on the metabolism of CAR T cells has been reviewed elsewhere (108). With the new understanding of T cell metabolism, researchers can further explore strategies to engineer CAR constructs that promote optimal metabolic profiles.

### 2.4 The impact of cytokines on CAR T cells

Furthermore, the metabolic state and differentiation of CAR T cells are heavily influenced by the cytokine milieu in which they operate. CAR T cells are cultivated and expanded in cytokine-enriched culture medium, with the final infusion product consisting mainly of T_eff_, T_em_ and T_cm_. In the stage of proliferation, they multiply their mitochondria and continue to rely on glycolysis to meet their increased energy demands. Currently, research predominantly focuses on utilizing cytokines IL-2, IL-7, IL-15, and IL-21. The ideal cytokine combination and the involvement of other cytokines in the production of CAR T cells remain undefined (109), primarily due to variability based on patient, tumor, and clinical context. IL-2 is the most frequently used cytokine in adoptive immunotherapy, mostly because it can significantly enhance the accumulation of CAR T cells and their cytotoxic capacity (109). IL-2 plays a crucial role in the differentiation of naive CD8^+^ T_n_ cells into T_eff_ and enhancing proliferation, together with upregulating perforin, granzyme B and IFN-γ while suppressing memory cell markers, such as CD62L, CCR7, CD27, BCL6 and IL7RA (110, 111). Additionally, IL-2 has been shown to favor Treg expansion, which can inhibit CAR T anti-tumor activity (112–114). IL-2 is also recognized as a promoter of T cell exhaustion in TME (115). To continue utilizing IL-2, Kaartinen et al. found that using lower doses resulted in favorable, less differentiated T cell population, but at the expense of reduced expansion and effector functions (116). One study investigated the effect of several cytokines, including IL-7, IL-15, and IL-21. They showed that these cytokines can reduce the CAR T cell’s terminal differentiation and increase the frequency of T_scm_, enhancing the effectiveness of adoptive immunotherapies. For example, IL-15 provided a less differentiated phenotype, with higher CD27 and CD28 expression, improving *in vivo* persistence and anti-tumor immunity in tumor-bearing hosts. Similar was shown for IL-7, which fostered the highest proportion of T_scm_, though its administration *in vivo* did not enhance anti-tumor effectiveness compared to IL-2 or IL-15 (109). Furthermore, the combination of IL-7 and IL-15 plays a crucial role in increasing the number of CD8^+^CD45RA^+^CCR7^+^ within the CAR T cell products, enhancing persistence and anti-tumor activity in preclinical models (78). Another promising cytokine is IL-21, which induced the expansion of less differentiated CAR T cells even after an antigen challenge. CAR T cells treated with IL-21 showed highest persistence in animal studies and led to more effective tumor elimination compared to most cytokines, except IL-15 (109). Recent study found that in the presence of antigen, IL-21-treated CAR T cells exhibited reduced glycolysis and enhanced OXPHOS, while markers of senescence were decreased compared to CAR T cells stimulated with IL-2 (117). Another strategy to counteract the immunosuppressive TME and to improve persistence and anti-tumor ability of CAR T cells, is to engineer CAR T cells to autonomously express immune-stimulating cytokines, such as IL-15 (118), IL-12 (119), IL-18 (120–123) and IL-23 (124). However, Zhao et al. demonstrated that also immunosuppressive cytokine IL-10 can metabolically reprogram CAR T cells by enhancing OXPHOS via mitochondrial pyruvate carrier-dependent manner, thereby promoting proliferation and effector functions. This reprogramming led to the complete regression of solid tumors in both syngeneic and xenograft mouse models. IL-10 CAR T cells also induced T_scm_ responses in lymphoid organs, providing durable protection against tumor rechallenge (125). First-in-human trial of Meta10–19 has demonstrated encouraging preliminary efficacy and a manageable safety profile with significantly higher complete response rate compared to commercial products (126). IL-10, traditionally viewed as an immunosuppressive cytokine, is now recognized for its immunomodulatory and metabolic-supportive roles in CAR T cell therapy, but requires further research to clarify downstream mechanisms enabling its diverse roles. To address the issue of CAR T cell exhaustion, Stewart et al. identified IL-4 as a key factor in CAR T cell dysfunction, separate from its established role in directing CD4^+^ CAR T cell polarization. Their research showed that IL-4 can induce exhaustion, marked by reduced T cell effector capabilities, increased expression of inhibitory receptors, and a transcriptional/epigenetic signature of exhaustion (127). Understanding and manipulating the cytokine environment could potentially enhance the efficacy and persistence of CAR T cell therapy and should be considered together with altering metabolic pathways.

### 2.5 Memory phenotype of CAR T cells and associated metabolism

**Figure 1:**
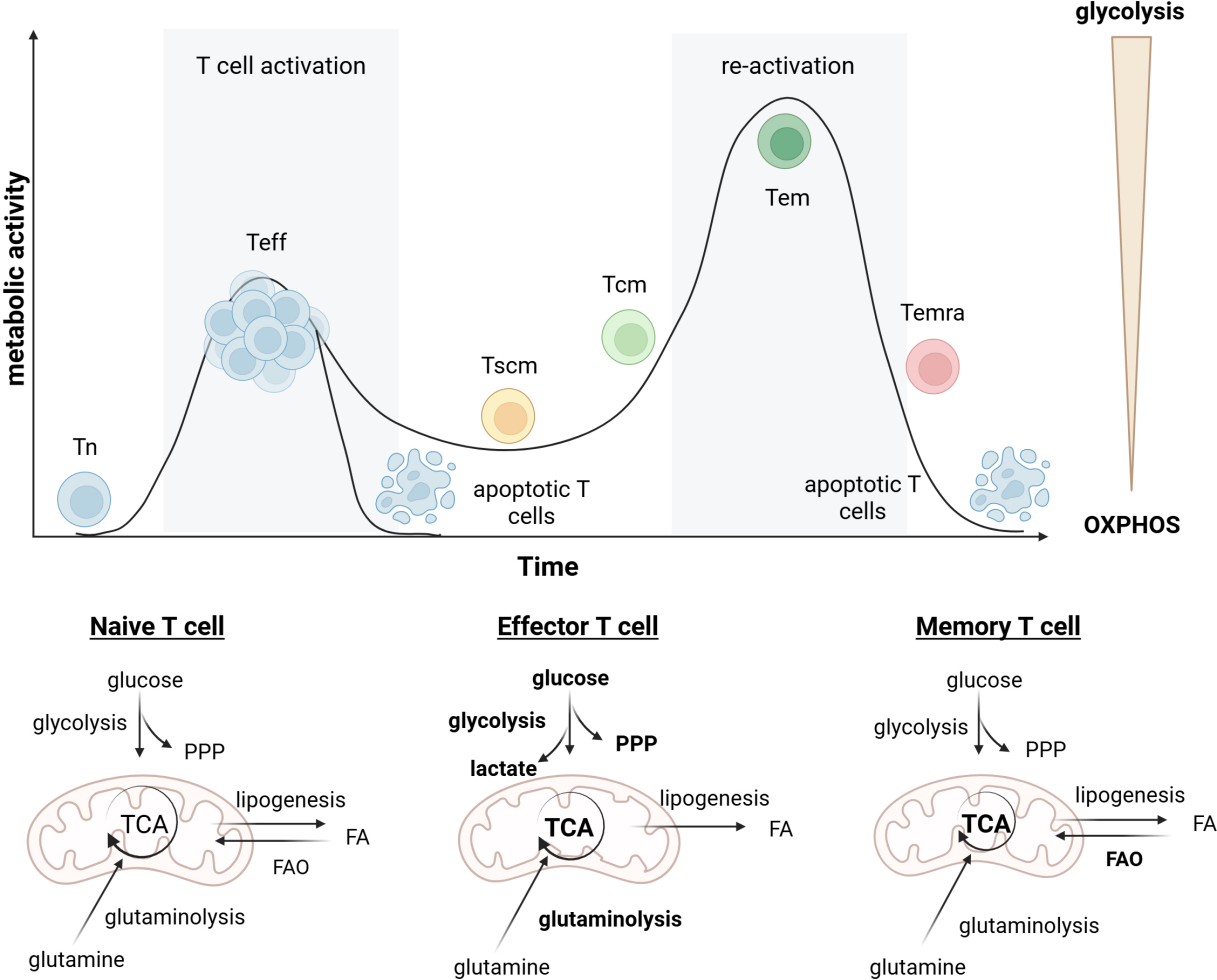
Metabolic programming of T cells during differentiation. T_n_ exhibit low metabolic activity and rely primary on OXPHOS. Upon antigen stimulation, T cells undergo activation and differentiate into T_eff_, characterized by a rapid increase in metabolic activity and a switch toward aerobic glycolysis and glutaminolysis to support proliferation and effector function. Following antigen clearance, most T_eff_ undergo apoptosis, while a small number of cells survive and transition into memory T cell subsets (T_scm_, T_cm_ and T_em_). Memory T cells display reduced glycolytic dependence and instead favor OXPHOS and FAO to sustain long-term persistence and rapid recall responses upon re-activation, while terminally differentiated T_emra_ exhibit diminished survival and metabolic activity.

Apart from T_eff_, a small subset of activated T cells acquires memory T cell properties and particularly this memory T cells in addition to T_n_ are crucial for efficient CAR T therapy. Four distinct subsets of memory T cells can be generally categorized into T_scm_, T_cm_, T_em_ and T_emra_ (128). Memory T cells have distinct metabolic demands, shifting to OXPHOS and favoring fatty acids for energy, enabling rapid, robust responses to subsequent antigen exposures (**Figure 1**) (129, 130). Memory CD8^+^ T cells formation is linked to AMPK-α1 signaling pathways, which facilitate FAO (131–133). Memory T cells also display lower baseline activity of anabolic signaling pathways such as PI3K-Akt-mTOR (130), do not rapidly proliferate, but instead generate energy to support their self-renewal and reside in secondary lymphoid organs (134). Importantly, memory T cells maintain substantial mitochondrial SRC and have increased mitochondrial mass compared to naïve and activated T cells (76).

Self-renewing memory T cells exhibit responses and protection upon re-exposure to antigens. This involves phenotypic changes, including upregulation of Bcl2 (anti-apoptic) (135), re-expression of CD62L/CCR7 and higher CD127 levels (136, 137). To form a stable pool of memory T cells, mTOR signaling must decrease post-initial immune response, and cells must adopt a non-glycolysis metabolic profile (130, 132, 138). Asymmetric distribution of mTORC1 enzymes in daughter cells affects differentiation; cells with more mTORC1 become effector cells, while those with less become memory T lymphocytes (139–141). Studies have also highlighted T-cell factor 1 (TCF-1) as an essential transcription factor for memory T cells, with Wnt-β-catenin regulating TCF-1 (142–144). Other transcription factors identified as memory T cell drivers include T-bet (T-box expressed in T cells) (145, 146), LEF-1 (Lymphoid Enhancer Factor-1) (147), Bcl-6 (B-cell lymphoma 6), FOXO1 (Forkhead Box O1) (148), Id2/3 (Inhibitor of DNA binding-2/3) (149), EOMES (eomesdermin) (145) and Blimp-1 (150, 151). The extended life span is partially due to IL-7/IL-15-dependent homeostatic proliferation (152).

One of the most important subpopulation for CAR T generation are minimally differentiated T_scm_ cells that constitute 2-3% of circulating T cells, found mainly in peripheral blood and secondary lymphoid organs (153). They share a CD45RA^+^CCR7^+^ phenotype with T_n_ but are distinct due to high CD95 expression (154, 155). They exhibit remarkable proliferative capacity, ability to self-renew, and have the potential to regenerate all other memory T cell populations (128, 156). Importantly, T_scm_ cells’ superior *in vivo* proliferation and survival correlate with enhanced anti-tumor efficacy compared to T_cm_ and T_em_ in mice (128, 142). Another subpopulation of T cells crucial for CAR T therapy and are ready T_cm_. They are more specialized, residing in lymphoid tissues and ready to rapidly expand and respond upon antigen re-exposure. While having limited or no effector function, they undergo efficient homeostatic proliferation and self-renewal (157). The transition from T_cm_ to T_em_ involves reduced expression of CCR7 and CD62L, resulting in diminished ability to migrate to lymphoid organs. T_em_ are progenitor cells prone to differentiate into T_eff_ cells upon secondary stimulation, are less proliferative, have decreased self-renewal and multipotency, but can migrate into inflammatory tissues for immediate response (158). Metabolically, T_cm_ cells rely more on FAO and OXPHOS for their proliferative and cytokine responses upon restimulation, while T_em_ cells have less dominant mitochondrial profiles (159, 160). T_scm_ exhibit decreased glucose metabolism and a preference for lipid metabolism, marked by high mitochondrial SRC, but are distinguished by a lower mitochondrial membrane potential (161). After extensive activation and differentiation due to repeated antigen exposure, T cells become terminally differentiated with decrease in mitochondrial mass and impaired energy production (162) (**Figure 1**).

## 3 Importance of T cell phenotype in CAR T cell therapy

**Figure 2:**
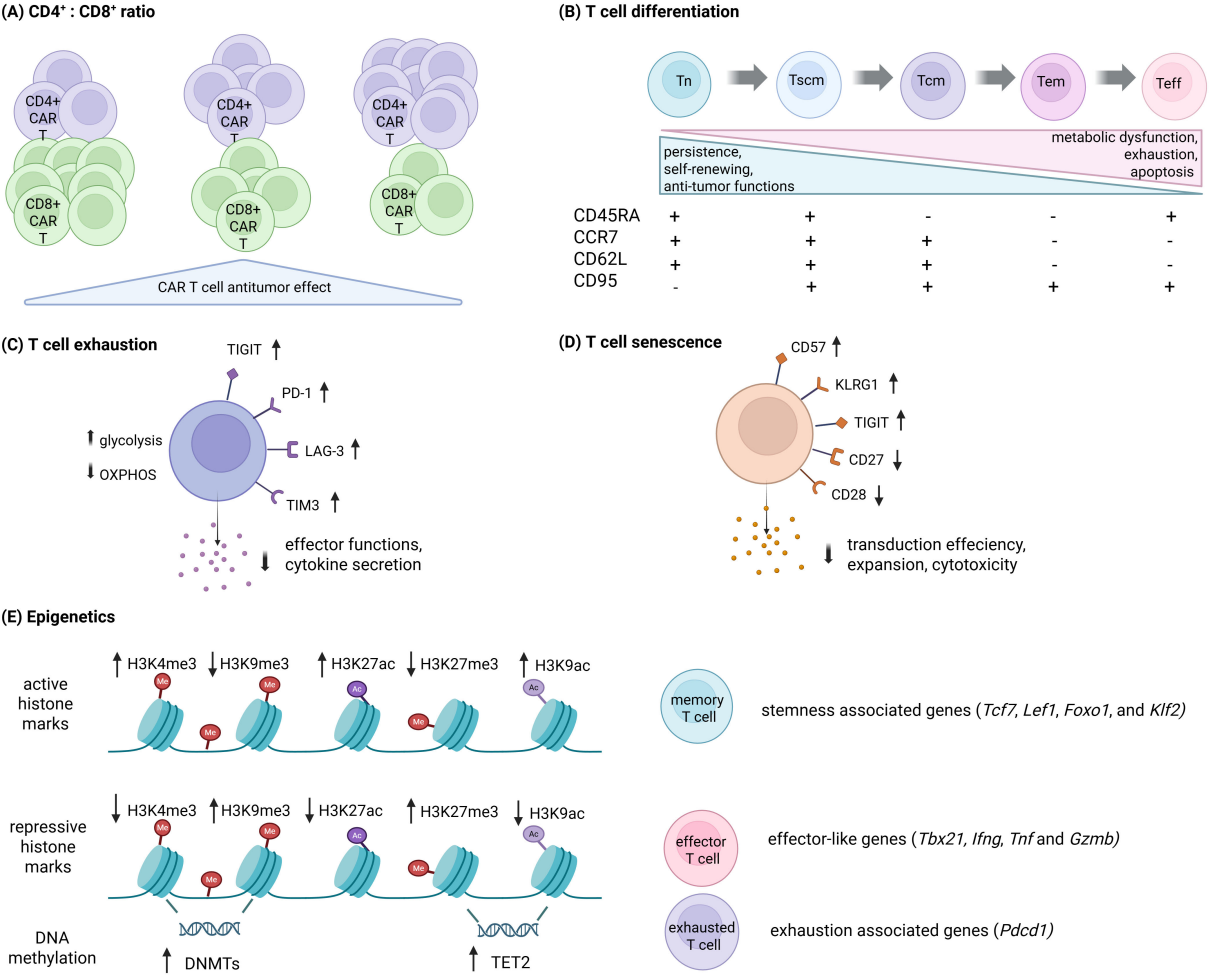
Factors influencing CAR T cell function and fate. **(A)** The CD4^+^/CD8^+^ ratio contributes to CAR T cell anti-tumor efficacy, with balanced subsets enhancing therapeutic outcomes. **(B)** T cell differentiation is associated with changes in persistence, self-renewal, and anti-tumor activity. Expression of surface markers (CD45RA, CCR7, CD62L, CD95) defines distinct subsets, where less differentiated T cells display superior persistence, self-renewing and anti-tumor functions, whereas terminally differentiated cells are prone to metabolic dysfunction, exhaustion and apoptosis. **(C)** T cell exhaustion is characterized by upregulation of inhibitory receptors (PD-1, TIM-3, LAG-3 and TIGIT), decreased glycolysis and increased OXPHOS, accompanied with impaired effector functions and reduced cytokine secretion. **(D)** T cell senescence involves increased expression of markers such as CD57, KLRG1, and TIGIT, along with decreased CD27 and CD28, leading to impaired transduction efficiency, expansion, and cytotoxicity. **(E)** Epigenetic regulation governs T cell fate, with histone modifications and DNA methylation controlling transcriptional programs. Active histone marks (H3K4me3, H3K9ac, H3K27ac) associate with memory gene expression (e.g. *TCF7, TBX21*), whereas repressive marks (H3K9me3, H3K27me3) and DNA methylation contribute to effector gene expression and exhaustion programs (e.g., *TOX, PDCD1*).

Therapy failure can be attributed to several factors, including elevated toxicity (163–166), antigen loss (165), and immunosuppressive TME (167). As discussed before, the suboptimal CAR T cell characteristics in the final product significantly contribute to this problem. Important factors to consider are CD4^+^/CD8^+^ ratio, T cell differentiation state, exhaustion, senescence and the epigenetic profile of the final CAR T cell product (**Figure 2**).

### 3.1 CD4^+^/CD8^+^ ratio

Recent research has indicated that the composition of T cell subsets in the apheresis product can be critical for the effectiveness of the final CAR T cell product. An appropriate CD8^+^ to CD4^+^ T cell ratio in the CAR T product is an important determinant of efficacy. Patients with B-cell malignancies show notable variations in CD8^+^ and CD4^+^ T cell subset proportions due to factors like age, pathogen exposure, or chemotherapy. While CD4^+^ CAR T cells produce more cytokines and exhibit robust proliferative capacity upon tumor encounter, CD8^+^ CAR T cells show increased lytic activity (168). A lower CD4^+^/CD8^+^ ratio was linked to better response rate of the patients (169), while multiple studies suggest an 1:1 ratio as the most beneficial in terms of anti-tumor effect (168–171). However, the expansion and function of CD8^+^ CAR T cells grown without CD4^+^ T cells should be considered (172).

### 3.2 T cell differentiation

CAR T cell products obtained by *ex vivo* expansion mainly comprise antigen-experienced T cells, predominantly T_em_ and T_cm_. The excessive rate of differentiation in the final product is a key factor contributing to the insufficient CAR T cell persistence and anti-tumor response in patients (78, 173–177). As already mentioned, high proportion of T_scm_ and T_cm_ in the final product is crucial for anti-tumor response (178). These two subpopulations have limited effector functions, however they can persist for a long time and can differentiate into T_em_ and T_emra_ with improved effector functions (179). Despite their high effector capacity, T_eff_ cells are considered less ideal for adoptive T cell therapy, primarily due to their short lifespan. Additionally, glycolysis-dependent T_eff_ face nutrient deprivation upon adoptive transfer, leading to significant cell death as their energy needs cannot be met (94, 95). Moreover, T_eff_ and T_emra_ exhibit higher degree of exhaustion, lacking replication or effector functions (180).

Conversely, T_cm_ and T_scm_ cells express high levels of homing receptors and persist well in lymphoid compartments, making them ideal for CAR T cell therapy, as they can traffic to secondary lymphoid organs and bone marrow. In addition, low differentiated T cells also demonstrate excellent anti-tumor responses and *in vivo* persistence due to strong self-renewal capacity. For example, CAR T cells generated by a homogenous T_n_ phenotype exhibit increased anti-tumor potential (181). In addition, CD19 CAR T cells derived from purified CD4^+^ or CD8^+^ T_cm_ or T_n_ were shown to exhibit improved metabolic adaptability and sustained anti-tumor activity (168). In a study on chronic lympholytic leukemia (CLL) patients, CAR T cells from complete responders showed memory-associated gene enrichment, while non-responders had upregulated effector differentiation, glycolysis, exhaustion, and apoptosis pathways (173). Similar findings were observed in patients with large B-cell lymphomas (LBCL) (174).

Many studies consistently link CAR T cell populations polarized towards a T_cm_-like phenotype with prolonged persistence and improved outcomes (182–186). In a study involving a patient with CLL who achieved complete remission following treatment with CAR T cells targeting the CD19 protein, it was observed that the CAR T cells originated from a ten-eleven translocation 2 (TET2)-targeted insertional mutagenesis event, exhibited T_cm_ phenotype at the peak of their expansion (187).

Following the discovery of T_scm_ population, studies demonstrated minimally differentiated T_scm_ as a population with superior persistence and anti-tumor response compared to T_cm_ (128, 142, 188). Furthermore, T_n_/T_scm_ demonstrated enhanced anti-tumor activity and capability to prevent leukemia recurrence in hematopoietic stem/precursor cell-humanized mice, exhibiting higher expansion, persistence and improved exhaustion/memory characteristics (189).

### 3.3 T cell exhaustion

T cell exhaustion, characterized by reduced T cell functionality due to persistent antigen stimulation during chronic infections and cancer, is a major hurdle. Recent evidence suggests that CAR structures themselves influence exhaustion, with higher tonic signaling from CAR self-aggregation causing CAR T cell exhaustion (190, 191). Exhausted cells exhibit reduced killing and proliferation, increased cell death, compromised metabolism, and heightened expression of inhibitory receptors such as programmed cell death protein 1 (PD-1), cytotoxic T lymphocyte-associated antigen 4 (CTLA-4), LAG-3 (lymphocyte activation gene 3), T cell immunoglobulin mucin receptor 3 (TIM-3), and T-cell immune receptor with immunoglobulin and ITIM domains (TIGIT) (98, 192, 193). Recently, Huang et al. have identified CD38 as another indicator of exhaustion, associated with suppressed glycolysis, which was co-expressed with exhaustion-related transcription factors and LAG-3 in anti-CD19 CAR T cells (194). Decreased expression of exhaustion markers on the surface of T lymphocytes prior to infusion has been shown to be an important predictor of therapy success (173, 174, 195). Less differentiated and less exhausted CAR T cells have been repeatedly shown to trigger stronger and more sustained anti-tumor responses, due to their longevity and proliferation rate (195–197). Correspondingly, higher frequencies of inhibitory receptors in leukapheresis T cells, peripheral or bone marrow T cells, or TME T cells correlate with worse responses in CAR T cell-treated patients (40, 195). It is also important to note that T cells from CAR T cell therapy candidates often have already severely impaired functions and metabolism due to previous treatments further negatively affecting therapeutic efficacy (198). Several principles have been tested to combat CAR T cell exhaustion in addition to CAR structure modification, such as anti-PD-1 antibody secreting CAR T cells (199), overexpressing the transcription factor c-Jun (200) and basic leucine zipper ATF-like transcription factor (BATF) (201) with the use of tyrosine kinase inhibitors (101). A recent study by Stewart and colleagues found IL-4 contributes to CAR T cell dysfunction and exhaustion; IL-4 neutralization enhanced anti-tumor effectiveness *in vitro* and *in vivo* while reducing exhaustion markers (127).

### 3.4 T cell senescence

T cell senescence involves the degeneration of innate and adaptive immunity, specifically the depletion of T_n_ and T_eff_ T cells during aging. As T cells approach the end of their lifespan, they can become senescent, leading to cell-cycle arrest while remaining viable and metabolically active (202). T cell senescence is distinct from T cell anergy and T cell exhaustion, which, despite having similar traits, originate from different causes. In replicative senescence, T cells lose co-stimulatory molecules like CD27 and CD28, while expressing markers such as CD57 and killer cell lectin-like receptor subfamily G (KLRG-1) (203). A recent study also suggested TIGIT as a novel T cell senescence marker (204). For CAR T cell therapy, senescent cells may significantly impact efficacy, but data are still limited. For example, an *in vitro* study comparing CAR T cells from young and elderly healthy donors concluded that CAR T cells from younger donors showed markedly higher transduction efficiency, expansion capabilities, and cytotoxicity (205).

### 3.5 Epigenetics

T cell functions are significantly influenced by epigenetic modifications, particularly DNA methylation and histone protein modifications (reviewed in (206, 207)). The term “epigenetics” refers to changes in gene expression, which are not caused by alterations in the DNA sequence but rather by post-translational modifications of DNA and surrounding histone proteins. Promoters and enhancers bind transcription factors, working with remodeling complexes and epigenetic modifiers to establish chromatin accessibility, which is either permissive or restrictive for gene expression. DNA methylation plays a role in preserving genome stability and is carried out by DNA methyltransferases (DNMTs), which suppress gene expression. Conversely, α-ketoglutarate (α-KG)-dependent ten-eleven translocation protein family (TET1-3) is responsible for DNA demethylation, thereby activating gene expression (208).

DNA is wrapped around histones, which undergo posttranslational modifications such as methylation, acetylation, ubiquitination, and lactylation. Histone modifications (H2A, H2B, H3, H4) are crucial for gene expression regulation. Histone methylation involves adding up to three methyl groups to lysine or arginine residues in the N-terminal tails of core histones H3 and H4, facilitated by histone methyltransferases (HMTs). Trimethylation of H3K4, H3K36, and H3K79 marks active transcription sites, while methylated H3K9, H3K27, and H4K20 are repressive marks (209). Histone acetylation plays a crucial role in chromatin remodeling and regulation of gene transcription, and is in general regarded as an activating histone modification (e.g., H3K27ac marks active promoters and enhancers). Histone acetylation is regulated by histone acetyltransferases (HATs), which transfer the acetyl group from acetyl-CoA to specific lysine residues of H3 or H4. Acetyl groups can be removed by histone deacetylases (HDACs), triggering chromatin recondensation.

Specific epigenetic profiles have been linked to complete response and overall survival in CAR T cell-treated patients (210). At the DNA level, TET2 is associated with upregulation of effector-associated genes (e.g., *Ifng*, *Tnf*, and *Gzmb*), while TET2 knockout (KO) promoted memory T cell formation with superior control upon secondary antigen encounter (211, 212). *Ex vivo* inhibition of TET2 also led to enhanced anti-tumor activity and proliferation of anti-CD19 CAR T cells in a patient with CLL (187). Also, 2-hydroxyglutarate, a competitive inhibitor of α-KG-dependent TET proteins, prolonged CAR T cell persistence (181), while α-KG treated CAR T cells showed increased tumor infiltration in fibrosarcoma-bearing mice (213). Furthermore, DNMT3A plays an important role in T cell effector differentiation, with *DNMT3A* disruption promoting memory phenotype through demethylation and re-expression of naïve cell-associated genes (214). In CAR T cells, the genetic deletion of *DNMT3A* preserved their differentiation capacity with sustained proliferation and effector function after repeated stimulation leading to superior control of tumor progression *in vivo* (215). Furthermore, CAR T cells have also been observed to experience a rapid and extensive removal of repressive DNA methylation programs at genes associated with effector functions, leading to a shift towards exhaustion-progenitor T cells (216). After extended antigen exposure, gene loci like *Pdcd1* exhibit *de novo* DNA methylation, stabilizing the exhausted phenotype (217). At the histone level, effector T cell transcription factor regulators show increased H3K27 trimethylation (H3K27me3), while memory T cells exhibit elevated H3K4 trimethylation (H3K4me3) and decreased H3K27me3 (137, 218). In particular, T_n_ and T_scm_ cells exhibit increased marks of H3K4me3 and reduction in H3K27me3 at key gene regions for *TCF7*, *LEF1*, *Foxo1*, and *Klf2* genes. Conversely, T_eff_-related genes and promoters (e.g., *Ifng*, *TBX21*) are linked to repressive histone marks (137, 147). Blimp-1 acts as an epigenetic regulator by directly recruiting repressive chromatin-modifying enzymes H3K9me3 and HDAC2 to Il2ra and Cd27 promoters, increasing short-lived effector cells while inhibiting memory precursor CD8^+^ T cell formation (219). KO of *PRDM1* (encoding Blimp1) helped preserve early memory T cells functional properties in human CAR T cells (220). Similarly, the histone methyltransferase SUV39H1, that catalyzes H3K9me3, plays a role in silencing memory-related genes during CD8^+^ T effector terminal differentiation (221). In CAR T cells, SUV39H1 genetic disruption improved memory transcription factor expression/accessibility and limited exhaustion after multiple re-challenges, correlating with early expansion, long-term persistence, and increased anti-tumor efficacy in leukemia and prostate cancer models (222). In addition, inhibition of enhancer of zeste homolog 2 (EZH2), a regulator of histone methylation, may also significantly enhance anti-tumor activity of CAR T cells (223).

Histone acetylation shapes differentiation trajectory and regulates CAR T cell exhaustion (219, 224–226). In particular, H3K27ac, which marks active gene expression, has been associated with memory T cell differentiation (227, 228). Zhu et al. recently highlighted HDAC inhibitors as a potential strategy to enhance CAR anti-tumor efficacy promoting better memory formation and resistance to exhaustion (229). Furthermore, studies demonstrate that acetyl-CoA, a substrate for histone acetyltransferase, mediates metabolism-coordinated transcriptional regulation through histone acetylation in T cells and CAR T cells (227, 228, 230, 231). Further research suggests that manipulating nuclear acetyl-CoA metabolism through enhancing nuclear-localized Acetyl-CoA synthetase 2 (ACSS2) or inhibiting ATP-citrate lyase (ACLY), to epigenetically reprogram terminal exhausted cells, improves anti-tumor responses (232).

## 4 Targeting metabolism to improve efficacy of CAR T cell therapy

A growing body of evidence highlights the critical importance of CAR T cells' metabolic state prior to infusion into patients (233, 234). Cellular metabolism has been recognized as a determining factor influencing T lymphocytes phenotype; differentiation, activation, effector properties and their longevity, rather than merely responding the changes in these states (76, 96, 235). It is crucial to recognize that altered cellular metabolism is not solely a consequence of T lymphocyte activation; instead, we can actively influence the differentiation and effector properties of T lymphocytes by modulating metabolism. As a result, by acting on the metabolism of T lymphocytes during *ex vivo* preparation of the cell product, the effectiveness of CAR T cell therapy can be improved, which is particularly important for highly exhausted T lymphocytes (235). The CAR T cell manufacturing process offers a unique opportunity to selectively expose these cells to drugs aimed at desirably altering their metabolism to stimulate long-term survival. Future research should prioritize the development of production strategies that foster favorable metabolic and epigenetic profiles, thereby generating CAR T cells with enhanced properties.

Despite significant advancements in CAR T cell therapy, primarily driven by sophisticated genetic engineering of CAR constructs (236), a critical need remains to deepen our understanding of the physiological characteristics of T cells during production and to optimize cultivation protocols. A more comprehensive grasp of the underlying biological mechanisms is essential for enhancing therapeutic outcomes.

As previously discussed, factors such as the CD4^+^/CD8^+^ ratio, cellular differentiation status, and exhaustion levels profoundly influence CAR T cell performance. Patient-specific variables (e.g. age, prior treatments) can alter the CD4^+^/CD8^+^ composition, with a balanced ratio shown to improve anti-tumor responses. Current *ex vivo* expansion protocols often produce metabolically fragile, terminally differentiated, glycolysis-dependent T_eff_ that are short-lived post-infusion. In contrast, T_cm_ and T_scm_ subsets demonstrate superior OXPHOS metabolism, persistence, and trafficking capabilities, which are associated with more durable clinical responses. T cell exhaustion—driven by chronic antigen exposure and CAR design— further compromises metabolic function and therapeutic efficacy. Additionally, senescence restricts proliferative capacity, particularly in older donors. Beyond metabolic adaptation, T cell functionality is also governed by epigenetic mechanisms which regulate the expression of metabolic genes. Recent studies highlight a strong interconnection between metabolic enzymes/substrates and epigenetic modifications (224, 225, 228), though these complex interactions warrant further investigation. Elucidating the interplay between metabolism, epigenetics, and T cell phenotype is crucial for refining CAR T cell manufacturing.

To address these multifaceted challenges, the following sections will explore targeted intervention strategies from three biological perspectives: (1) inducing a memory phenotype with metabolic modulators; (2) leveraging metabolites as signaling molecules to remodel the epigenetic landscape; and (3) directly modulating the nutrient environment to optimize the cellular metabolic state.

These three perspectives represent a shift from purely structural CAR design toward a more holistic approach centered on the T cell’s functional state. Inducing a memory phenotype through metabolic reprogramming— suppressing glycolysis and enhancing OXPHOS—achieves greater persistence, reduced exhaustion, and improved anti-tumor activity, aligning with the metabolic robustness observed in memory-like subsets T_cm_ and T_scm_.

Concurrently, the use of metabolites as signaling entities offers a powerful means to influence the epigenetic landscape of CAR T cells. Metabolites like acetyl-CoA, α-KG, and S-adenosylmethionine serve as cofactors for chromatin-modifying enzymes, linking metabolic flux to gene expression programs. Modulating these pathways during manufacturing may enable the imprinting of favorable transcriptional states that support long-term functionality and resistance to exhaustion.

Finally, direct manipulation of the nutrient environment through supplementation/restriction of key substrates such as glucose, glutamine, and fatty acids offers a practical avenue to fine-tune the metabolic state, influencing signaling, differentiation, and survival prior to infusion. This paper highlights the novel implications of metabolic modulations and their potential to alter the CAR T cell characteristics during manufacturing, resulting in a less exhausted, memory-like phenotype with enhanced effector functions, which could significantly improve CAR T therapy (Summarized in **Table 2**).

**Table 2:** Metabolic modulators rewiring CAR T cell metabolism.

Inhibitor	Target	Mechanism	Impact on CAR T cell therapy	Ref.
2DG	Hexokinase	Glycolysis inhibition; preventing N-glycosylation of proteins in ER	Increased formation of memory CTL019 lymphocytes with enhanced proliferative capacity	(173)
LY294002, IC87114, MK2206, TGR-1202	PI3K	Blocking the PI3KAKT-mTOR signaling pathway	Increased T_n_/T_scm_ and T_cm_ populations, decreased T_eff_ population	(237, 238)
Duvelisib	PI3Kδ/γ	Blocking the PI3K-AKT-mTOR signaling pathway	Enhanced number of T_scm_ and T_n_, normalized CD4/CD8 ratios, increased mitochondrial mass, and epigenetic modifications	(239)
Idelalisib	PI3Kδ	Blocking the PI3K-AKT-mTOR signaling pathway	Less-differentiated phenotype, decreased PD-1 and TIM-3, improved transduction efficiacy and cytotoxicity	(240–242)
Rapamycin	mTOR	Regulates glycolysis and glucose transport	Promoting memory phenotype, improvement in *in vivo* reporter and CAR gene transfer rates, increased the capacity of CAR T cells to infiltrate bone marrow	(25, 243, 244)
NCI-737	LDH	Reversibly inhibits the activity of the LDHA and LDHB isoforms of LDH	Stimulate the development of T_scm_ CD8^+^ cells, enhancing OXPHOS, preventing lactate production	(245)
Oxamate	LDHA	LDHA inhibition	Reducing tumor-infiltrating CAR-Treg cells	(246)
AZD3965, AR-C155858	MCT-1	Inhibition of lactate transport	Improved cytotoxicity *in vitro* and anti-tumoral control on mouse models	(247)
DON	Glutamine	Glutamine antagonist	Stimulate OXPHOS and FAO, with suppression of the glycolytic activity	(248, 249)
Mdivi-1	Mitochondria	Fission inhibitor	Impacting cristae structure, which guides the development od memory T cells	(97)
Urolithin A	PGC1α	Mitophagy inducer	Promoting T_scm_ formation in *ex vivo* cultured CAR T cells	(250)
Semaglutide	GLP 1R agonist	Autophagy induction through mTOR activation	Increased CD8^+^ T memory cells, enhancing persistence and anti-tumor activity *in vitro* and in xenograft models in synergy with Urolithin A	(251)
Spermidine	/	Autophagy inducer	Lower differentiation degree of CAR T cells	(252, 253)
Bezafibrate	PGC-1α	Agonist of PGC-1α/PPAR complexes	Significant expansion and enhanced anti-tumor response of CAR T cells *in vivo*	(254)
UK5099	MPC	Epigenetic modifications	Superior and long-lasting anti-tumor activity	(228)
MITO-66	MPC	Epigenetic modifications and metabolic compensation	Increased T_scm_ number with improved anti-tumor functions of CD19 CAR T cells	(255)
Enasidenib	IDH2	Diverting glucose utilization into the PPP, blocking the conversion of isocitrate to α-KG	Reducing CAR T cell exhaustion, improved memory T cell signature, tumor eradication and CAR T cell persistence *in vivo*	(227)
Metformin	ETC	Gluconeogenesis inhibition, AMPK activation, boosting catabolic processes	Increased proportions of T_scm_ and T_cm_ cells with enhanced anti-tumor capabilities, increased oxidative metabolism	(256, 257)
Nicotinamide mononucleotide	NAD^+^	NAD^+^ –Sirt1 axis	Increased T_scm_ and T_cm_ subpopulations of CD19 CAR T cells, enhanced mitochondrial activity of T cells from young donors	(258, 259)
Nicotinamide riboside	NAD^+^	enhanced NAD^+^ availability and sirtuin activation	Preventing TOX upregulation upon chronic *in vitro* stimulations in young adults	(258)
Avasimibe	ACAT1	Modulating cholesterol esterification	Heightened cytotoxic effect of CD19 directed CAR T cells on K562 target cells	(260)
Resveratrol	Sirt1	antioxidant activity'	Decrease in activation of T cells in case of severe toxicity in primary human T cells and a leukemia xenograft mouse model	(261)

### 4.1 Metabolic modulators

**Figure 3:**
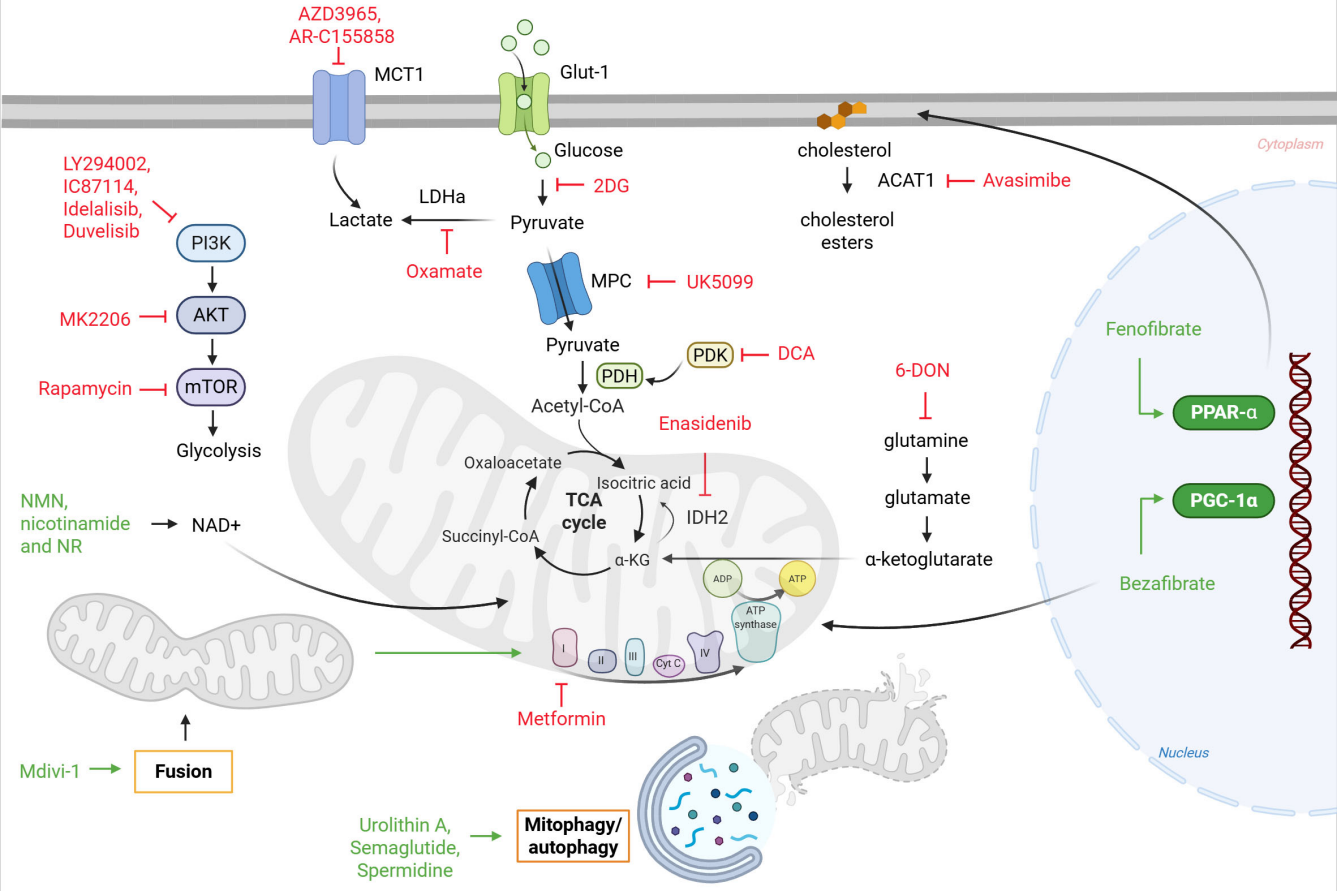
Targeting metabolic pathways to modulate CAR T cell function. Key metabolic processes in T cells can be pharmacologically regulated to enhance or restrain function. TCR signaling through CD28 and CD3ζ activates the PI3K–AKT–mTOR pathway to promote glycolysis, which can be inhibited by LY294002, IC87114, Idelalisib or Duvelisib (PI3K inhibitors), MK2206 (AKT inhibitor) and Rapamycin (mTOR inhibitor). Glucose uptake via GLUT1 and subsequent glycolysis can be blocked by 2-deoxyglucose (2DG), lactate production by Oxamate and its transport through MCT1 by AZD3965 and AR-C155858. Pyruvate entry into mitochondria via MPC can be inhibited by UK5099, whereas PDH activity is regulated by PDK, which can be inhibited by DCA. TCA cycle flux is further influenced by IDH2, targeted by Enasidenib. Glutamine metabolism to α-ketoglutarate is inhibited by 6-DON. In ETC, Metformin inhibits mitochondrial complex I, thereby reducing OXPHOS. Cholesterol esterification via ACAT1 can be blocked by Avasimibe, while activation of PPAR-α and PGC-1α enhances mitochondrial biogenesis and FAO. Additional strategies include enhancing NAD^+^ availability (NMT, nicotinamide and NR), promoting mitochondrial fusion (Mdivi-1), and stimulating mitophagy (Urothilin A, semaglutide and spermidine), all of which contribute to sustaining CAR T cell metabolism and function.

To date, several metabolic modulators have been under investigation in the context of improving CAR T cell persistence and anti-tumor properties. These metabolic modulators target various pathways and mechanisms involved in T cell metabolism and function, which are shown in **Figure 3**.

#### 4.1.1 Glycolysis inhibitors

As already mentioned, increased glycolytic activity in T cells can accelerate their terminal differentiation, while reducing glycolysis supports memory CD8^+^ T cell formation (134). The most studied glucose metabolism inhibitor is 2-Deoxy-D-glucose (2DG), a glucose analog that enters cells via GLUT transporters and is phosphorylated by hexokinase but cannot be further metabolized, thus inhibiting hexokinase. 2DG also restricts mannose incorporation into N-glycosylation pathways, potentially causing endoplasmic reticulum (ER) stress and the unfolded protein response (UPR) (262–264). In addition to glycolysis, targeting N-glycosylation has also been shown to improve exhaustion and CAR T cells efficiency in xenograft models of solid tumors (265). 2DG is explored as a potential anti-cancer drug (266, 267) and has shown promise for improving adoptive T cell therapy *ex vivo*. However, high 2DG concentrations (> 1 mM) may suppress anti-tumor T cell responses by reducing IFN-γ secretion and energy metabolism (71, 268). Some studies using lower concentration of 2DG (1 or 2 mM) showed minimal proliferation inhibition while successfully inducing OXPHOS and a shift to memory phenotype (99, 173). Recently, our group demonstrated that low dose 2DG (0.15–0.6 mM) treatment of pre-activated T cells significantly increased IFN-γ production, reduced PD-1 and increased CD69 expression, while the concentration above 2.4 mM 2DG had an opposite effect. However, the mechanism of this dual effect of 2DG remains unknown (264). Another study showed that 2DG could also sustain FOXO1 activity and enhance lymphoid homing (99). In CAR T cells, 2DG-mediated glycolysis inhibition decreased the differentiation into effector cells and increased the frequency of central memory CAR T cells. Specifically, blocking glycolysis through pharmacological means simultaneously encouraged the development of memory CTL019 lymphocytes, which exhibited an increased ability to proliferate following re-exposure to tumor cells expressing CD19 (173). Thus, studies indicate that inhibition of glycolysis and N -glycosylation using 2DG in appropriate dosage and timing could be beneficial in adoptive therapies such as CAR T therapy, however, the exact mechanisms through which 2DG is affecting T cell phenotype remain to be explored and different results depending on the treatment timing warrant some caution and further investigation.

#### 4.1.2 LDH inhibitors

Lactate dehydrogenase (LDH), specifically LDH-A, is a key metabolic enzyme converting pyruvate to lactate and NADH to NAD^+^. It is also a critical regulator of effector differentiation and function across immune cells (Reviewed in (269)). TCR signaling induces LDH-A expression through PI3K/Akt signaling in activated T cells promoting lactate production. In CD4^+^ cells LDHA deficiency resulted in a defective CD8^+^ T-cell expansion and differentiation by impairing the Akt and FOXO1 phosphorylation (59, 270, 271). LDH-A was also previously shown to promote IFN-γ expression in T cells through a high levels of acetyl-CoA to enhance histone acetylation (272). However, LDH inhibition can inhibit terminal effector and exhaustion programs through promoting the pyruvate entry into the TCA. LDH-A inhibition combined with IL-21 *in vitro* promoted the formation of T_scm_ with increased anti-tumor activity *in vivo* after adoptive transfer (245). The impact of lactate metabolism on CAR T cell function is not well understood, with few studies exploring the inhibition of LDH in order to improve CAR T cells *ex vivo* (246, 271). Combining CAR T cells with LDH-A depletion notably delayed tumor growth and increased survival in prostate cancer mice (271). Additionally, Oxamate, an LDH-A inhibitor and analog of pyruvate, altered the phenotypes of tumor-infiltrating CAR T cells in GBM mouse model with increased expression of effector molecules. Oxamate also alleviated TME immunosuppression and reduced tumor-infiltrating CAR- regulatory T cells (Treg) cells by downregulating CD39, CD73 and CCR8 levels (246). Beyond LDH inhibitors, blocking lactate transport via monocarboxylate transporter (MCT)-1 with AZD3965 or AR-C155858, improved CAR T therapy against B-cell malignancies (247). Manipulating lactate metabolism thus holds promise for improving CAR T cell function in solid tumors with immunosuppressive environments and for reprogramming T cell metabolism *in vitro*.

#### 4.1.3 PI3K/Akt/mTOR inhibitors

Activation of the PI3K pathway in T cells is initiated by TCR signaling and facilitates the transmission of co-stimulatory signals through CD28. The PI3K/Akt/mTOR signaling pathway is vital for T cell activation, proliferation, differentiation, and survival across many cell types. Sustained activation of PI3K/AKT/mTOR from persistent stimulation (e.g., with beads, IL-2, or tonic CAR signaling) promotes terminal differentiation of CD8^+^ T cells via mTOR-mediated effects on glycolysis, making PI3K and AKT an attractive target for modulation during anti-CD3/CD28 stimulation (273). Long et al. showed that CD28 costimulatory domain increased CAR T cell exhaustion through persistent CAR T signaling, while 4-1BB had less effect (190). Within CAR T cells, CD28 induces signaling including activation of PI3K/AKT/mTOR pathway. Although PI3K signaling enhances proliferation, excessive signals may lead to T cell exhaustion and terminal differentiation, correlating with suboptimal clinical responses. Therefore, PI3K/Akt/mTOR inhibitors may have significant importance in suppressing rapid differentiation and glycolysis driven by CD28 signaling (274). Earlier studies have demonstrated that adoptively transferred T cells following *ex vivo* AKT inhibition display enhanced anti-tumor efficacy (275), preserved memory-like characteristics, increased cytotoxicity and expansion (276–278). The addition of PI3K inhibitors (LY294002 or IC87114) during *ex vivo* CAR T cell expansion increased memory populations and decreased the T_eff_ populations of CD33 CAR T *in vivo*, resulting in improved persistence and cytokine production (237). Linda et al. also found that PI3Kδ inhibition with LY294002 regulates the differentiation and function of T cells *in vitro* and in mouse experiments (240). Furthermore, the addition of Akt inhibitor MK2206 maintained CAR T cells in a less differentiated state *in vitro* without impairing their activation, leading to significantly longer CAR T cell persistence and enhanced anti-tumor activity *in vivo* (276).

In addition, Duvelisib and Idelalisib are FDA-approved drugs that also show potential for improving CAR T therapy, with Idelalisib as the first dual-PI3Kδ/γ inhibitor to be approved for treating r/r CLL (241). PI3K-δ is constitutively expressed in hematologic malignancies, and its inhibition reduces the proliferation of various hematologic tumor cells while allowing normal immune cells to survive (279). Adding dual-PI3Kδ/γ inhibitor Duvelisib during CAR T cell manufacture yields cells with an enhanced number of memory subsets, normalized CD4/CD8 ratios, increased mitochondrial mass, and epigenetic modifications correlating with enhanced anti-tumor efficacy (239). Duvelisib also shows promise for treating CAR T-associated cytokine release syndrome (CRS) clinically (280). However, T cells co-inhibited with both PI3Kγ and PI3Kδ inhibitors were functionally impaired, showing reduced effector cytokine production post-antigenic re-encounter and decreased *in vivo* persistence. Idelalisib, another PI3K inhibitor that targets PI3K-δ, was described to improve CAR T cell products, particularly when derived from CLL patients (242), and anti-mesothelin CAR T cells (281). In CAR T cells from CLL patients, Idelalisib induced less-differentiated phenotype (CD45RA^+^CCR7^+^CD62L^+^) with decreased expression of exhaustion markers (PD-1 and TIM-3) together with improved transduction efficacy without affecting viability and proliferation (242). Furthermore, expansion of T cells in the presence of Idelalisib significantly enhanced their anti-tumor activity also *in vivo* in a xenograft mouse model and a murine lymphoma model (242, 282). Altogether, sole blockade of PI3Kγ or PI3Kδ isoforms generates T cells with remarkable anti-tumor properties, with substantial clinical implications (283).

In addition, studies have demonstrated that the suppression of the mTOR pathway through the administration of Rapamycin, EMA- and FDA-approved inhibitor of mTOR, leads to the formation of CD8^+^ memory T cells (132, 284). In addition, expanding CAR T in the presence of Rapamycin promoted a T_scm_ phenotype (25). Administering Rapamycin to PBMC-humanized mice before injecting vectors, led to a significant improvement in both *in vivo* reporter and CAR gene transfer rates (243). Rapamycin pretreatment furthermore increased the capacity of CAR T cells to infiltrate bone marrow and enhanced the extent of bone marrow AML cell elimination in leukemia xenograft mouse models (244). Together with metformin, Rapamycin treatment of glioma mice before CAR T cell therapy also enhanced ATP production in the mitochondria, leading to better outcomes in terms of both effectiveness and survival (285). One group developed Rapamycin-induced caspase 9 suicide gene, and co-expressed it with CD19 CAR to induce safety switch designed to selectively eliminate CD19 targeting CAR T cells in case of adverse effects (286). One recent study also investigated the role of Rapamycin in CAR-modified hematopoietic stem cell therapy with HIV infected humanized mice, where Rapamycin reduced persistent immune activation improving CAR T cell function and cytotoxicity. They have also reported improved mitochondrial function with decreased exhaustion and increased stemness-related gene expression (287). Rapamycin is therefore a well-characterized drug with known pharmacokinetics, which could help to prolong CAR T cell functions, improve gene transfer and minimize adverse immune reactions.

#### 4.1.4 Glutamine antagonists

Glutamine metabolism is indispensable for effector T cell differentiation and function (288). Glutamine deprivation was reported to reduce T cell viability and proliferation (289), while glutamine depletion was able to increase the proportion T_cm_ upon activation of naive CD8^+^ T cells in tumor-inoculated mouse model (290). Furthermore, short-term *ex vivo* exposure of CAR T cells to glutamine-restricted conditions increased their *in vivo* persistence, enhanced the Th1 immune response, and improved cytotoxic CD8^+^ T lymphocyte effector function (291). Recently, genetically programmable nanoparticles infused with glutamine antagonists have been developed to target tumor sites. This approach significantly reduces immunosuppressive cells and boosts anti-tumor efficacy and long-term memory immunity of CAR T cells, which could be particularly beneficial for solid tumors (292). Glutamine antagonist 6-Diazo-5-oxo-l-norleucine (DON), a structural analogue of glutamine, inhibits a broad range of glutamine-requiring enzymes. DON decreases the reliance of T cells on glutamine metabolism but does not completely impede the uptake and breakdown of glutamine. Tumor-infiltrating CD8^+^ T cells extracted from mice and treated with the prodrug of DON enhanced anti-tumor activity by promoting highly activated, memory subtypes (248). In CAR T cell manufacturing process, *ex vivo* DON exposure can stimulate OXPHOS and FAO while suppressing glycolysis, leading to higher proportion of T_n_ or T_cm_ subsets with enhanced anti-tumor effect *in vitro* and *in vivo* (249).

#### 4.1.5 Modulators of mitochondrial fission/fusion

Mitochondrial remodeling is a signaling mechanism that instructs T cell metabolic programming. In memory T cells, mitochondrial fusion modifies cristae structure to support OXPHOS and FAO, whereas fission in T_eff_ cells causes cristae enlargement, reducing electron transport chain (ETC) efficiency and promoting aerobic glycolysis (97). Therefore, altering the cristae of mitochondria through fusion/fission instructs metabolic adaptations in T cells and can be modulated to enhance anti-tumor immunity. Data suggests that fusion-promoting (293) or fission-inhibitory drugs (294) create more metabolically fit T cells. For example, mice receiving fusion-promoted drugs were able to control tumor growth significantly better than control-treated mice. In activated human T cells, Mdivi-1 treatment *in vitro* induced mitochondrial fusion and exhibited bioenergetic profile and surface marker expression characteristic of memory cells (97). Authors speculated that fission-associated expansion of cristae due to TCR stimulation physically separates ETC complexes, decreasing efficiency. Conversely, when cristae are compactly organized, ETC operates at peak efficiency, promoting the influx of pyruvate into the mitochondria and maintaining a favorable redox state. Under these conditions, the fusion-induced cristae structure guides the development of memory cells and maintains them in a quiescent state (97). In CAR T cells, Mdivi-1 was tested as CD39 modulator, with intention to investigate the role of CD39 in the functional performance of CAR T cells against hepatocellular carcinoma. Mdivi-1 was able to increase the frequency of CD39 intermediate-phenotype CAR T cells, improving cytokine secretion, proliferation, and cytotoxicity, but also increased apoptosis and exhaustion rates. Specifically, the combination of Mdivi-1 and CD39 knockdown demonstrated a robust anti-tumor activity *in vivo* (295).

#### 4.1.6 Mitophagy/autophagy inhibitors

Beyond regulating mitochondrial function, mitophagy is critical for removing damaged or dysfunctional mitochondria and maintaining the anti-tumor response of CD8^+^ T cells within the TME (296). Since mitochondrial accumulation characterizes exhausted and dysfunctional tumor-infiltrating lymphocytes (TILs), inducing mitophagy could enhance anti-tumor immunity (296). Urolithin A, a natural metabolite of elagitannins, of which pomegranates present a main source, was recently found to induce Pink-1 dependent mitophagy and stimulate compensatory mitochondrial biogenesis by up-regulating PGC1α, thereby promoting T_scm_ formation in *ex vivo* cultured CAR T cells (250). In line with that, another study found that Urolithin A can promote memory formation, improve persistence and enhance the anti-tumor functions of human CAR T cells. Intriguingly, Urolithin A enhanced TCR signaling and activated NFAT, leading to increased production of autocrine IL-2 and effector molecules. Forming activating NFAT-AP-1 complexes is vital for preventing T cell exhaustion, enhancing their long-term ability to proliferate, and supporting effective anti-tumor responses (200). Urolithin A also directly binds ERK1/2 kinases, enhancing their activation and activating autophagy initiator ULK1 to facilitate autophagy. Specifically, the UA-ERK1/2-ULK1 cascade reduces oxidative stress and enhances cellular metabolism (297). However, these studies have not examined the effect of Urolithin A under multiple tumor re-challenges. Akhtar et al. observed that CAR T cells undergo metabolic alterations during tumor re-challenge with accumulation of dysfunctional mitochondria and potential impairment in autophagy process, which was also correlated with defect in memory CAR T cell formation. Screening autophagy/mitophagy-promoting molecules, they identified GLP 1R agonist (Semaglutide) and Urolithin A as the most promising candidates. Semaglutide and Urolithin A synergistically eliminated dysfunctional mitochondria with Semaglutide inducing autophagy through mTOR activation, and Urolithin A activating Atg4b. These changes increased the proportion of CD8^+^ T memory cells, enhancing persistence and anti-tumor activity *in vitro* and in xenograft models. They further developed metabolically reprogrammed CAR T cells with enhanced autophagy and mitophagy (251). This suggests targeting autophagy/mitophagy as a possible approach to improve CAR T cell therapy, requiring additional clinical research. Another autophagy inducer is spermidine, a polyamine compound that regulates human metabolism, possesses anti-aging potential (298), and promotes lymphocyte generation (299). Spermidine has been demonstrated to enhance the formation of memory CD8^+^ T-cells in aged mice following vaccination, resulting in improved memory function (252). *In vitro* treatment of nanobody-based (Nb) CAR T cells with spermidine provided lower levels of differentiation, with T_cm_ accounting for more than 55% of the total population. Importantly, spermidine promoted Nb CAR T mediated cytotoxicity to lymphoma cells by enhancing memory subsets and proliferation. Post-co-incubation with Daudi cells, IL-2, IFN-γ and LDH were significantly increased in spermidine pretreated groups. In an *in vivo* hematologic tumor model, spermidine pretreated groups showed reduced tumor burden and improved mouse survival (253).

#### 4.1.7 Modulators of mitochondrial biogenesis

PPAR-γ coactivator-1α (PGC-1α) is a key regulator of mitochondrial biogenesis, which leads to metabolic reprogramming and enhanced T cell-mediated anti-tumor immunity (300, 301). PGC-1α and its cofactors induce transcription factors activating FAO, OXPHOS, and mitochondrial expansion, which promotes CD8^+^ activation and proliferation (301). Bezafibrate, aPGC-1α/PPAR agonist can increase or maintain the number of functional CD8^+^ T cells by activating mitochondrial and cellular metabolism (302). Bezafibrate showed synergistic tumor-suppression activity with anti-PD-L1 monoclonal antibody (mAb) in mouse colon xenograft models (301, 302). In CAR T cells, a modified form of PGC-1α, stimulated mitochondrial biogenesis and metabolically reprogrammed CAR T cells, resulting in improved anti-tumor efficacy in solid tumors (303). Wang et al. demonstrated that *PRDM1* deficient CAR T cells showed increased levels of PGC1α and displayed improved proliferation and memory development, as well as resistance to exhaustion after multiple re-challenges. Improved CAR T cells expansion and persistence was also achieved *in vivo*. Importantly, the use of Bezafibrate in CAR T cells led to more significant expansion of CAR T cells and suppressed tumor growth without noticeable side effects (254).

#### 4.1.8 MPC inhibitors

Import of pyruvate across the inner mitochondrial membrane requires a specific carrier, named mitochondrial pyruvate carrier (MPC), a complex of two proteins (MPC1 and MPC2), serving as the exclusive gateway for pyruvate to enter into mitochondria (304). Mitochondrial pyruvate import is also essential for thymic T cell precursor development (305). Wenes et al. demonstrated that UK5099 inhibitor, targeting MPC in mouse CAR T cell, resulted in chromatin accessibility at pro-memory genes and thus memory differentiation orchestrated by RUNX1. They showed that CAR T cell manufacturing with an MPC inhibitor led to superior and long-lasting anti-tumor activity. Genetic deletion of MPC further proved specificity, not affecting effector functions but skewing CD8^+^ differentiation towards a memory phenotype. However, MPC deletion in a nutrient-deprived TME blunted CD8^+^ T cell effector function due to inability to oxidize lactate in mitochondria (228). The same research team subsequently employed a new MPC inhibitor, MITO-66, which effectively triggered a stem cell-like memory phenotype and improved anti-tumor effectiveness of CD19 CAR T cells from healthy donors and r/r B cell malignancy patients, facilitating clinical application (255).

#### 4.1.9 Dichloroacetate

Dichloroacetate (DCA), a generic medication used in humans for over three decades, has molecular mechanisms that are still largely unknown. Through the suppression of phosphoinositide-dependent kinase-1 (PDK1), it directs pyruvate toward TCA cycle, shifting the metabolism from glycolysis to mitochondrial glucose oxidation (306). Ohashi et al. found DCA-treated tumor cells improved T cell dysfunction caused by tumor-secreted lactic acid. DCA enhanced immune function and anti-tumor immune-reactivity by manipulating glucose metabolism in tumor cells, leading to increased T cell proliferation and cytokine production, with diminished apoptosis (307, 308). In human alloreactive lymphocytes, DCA inhibits aerobic glycolysis and induces Treg differentiation (309). Moreover, DCA significantly improves anti-tumor immune responses, resulting in increased anti-tumor efficacy and extended survival in mouse models of both ascitic and subcutaneous HCC (310). In an *in silico* screen, Renauer et al. identified PDK1 as metabolic regulator of CAR T cell functionality (311). Therefore, more studies are needed to better understand the role of PDK1 in CAR T therapy or potentially use DCA in CAR T cells targeting solid tumors where acidic microenvironment presents a major barrier.

#### 4.1.10 Enasidenib

IDH2 plays a central role in the TCA cycle, catalyzing the conversion of isocitrate to α-KG. Recently, isocitrate dehydrogenase 2 (IDH2) was identified as the regulator of memory T cell differentiation of murine T cells via reductive carboxylation of α-KG, and IDH2 inhibition also enhanced CAR T cell functionality (312). Si et al. tested Enasidenib, an IDH2 inhibitor and an FDA-approved clinical drug for leukemia. Enasidenib impedes metabolic fitness of CAR T cells by diverting glucose utilization into the pentose phosphate pathway (PPP), which alleviates oxidative stress and reduces CAR T cell exhaustion, particularly in nutrient-restricted conditions. However, CAR T cell memory formation is thought to be regulated through additional mechanism. IDH2 inhibition resulted in accumulation of isocitrate and citrate in CAR T cells, suggesting an overall blockade of IDH-mediated oxidative decarboxylation. This enables citrate to freely shuttle between the cytosol and nucleus and supports acetyl-CoA levels, which is a main epigenetic mediator of histone acetylation. In CAR T cells, the level of H3K27ac was notably elevated following the inhibition or removal of IDH2, and the H3K27ac-regulated genes indicated the expression of a memory T cell signature. In combination with pharmacological IDH2 inhibition, CAR T cell therapy is associated with improved tumor eradication and CAR T cell persistence *in vivo* (227). This research underscores the potential to enhance CAR T cell effectiveness by modulating their metabolism, particularly acetyl-CoA levels, which can reprogram their epigenetic profile, offering a new perspective on improving their function.

#### 4.1.11 Inhibitor of electron transport chain (ETC) - metformin

Metformin is a standard first line antidiabetic drug, acting systemically by inhibiting gluconeogenesis in the liver and improving glycemic control. It can also act directly by inhibiting complex I of the respiratory ETC, which leads to decreased OXPHOS ATP production and decreased ATP/AMP ratio, leading to AMPK activation. This suppresses anabolic and boosts catabolic processes. Metformin is a pleirotropic compound and surprisingly, its mechanisms are still not fully understood. In the recent years, metformin has received a lot of attention as a potential anti-cancer agent (313, 314). It has been found to directly normalize or improve CD8^+^ T cells anti-tumor capacity. Finisguerra et al. showed metformin could increase CD8^+^ T cell fitness in hypoxia, both *in vitro* and *in vivo* (315). Metformin could also enhance FAO of T cells by stimulating AMP-dependent kinases and increasing differentiation towards memory phenotype. Metformin treated HER2 CAR T cells showed increased proportions of T_scm_ and T_cm_ cells, along with enhanced anti-tumor capabilities, likely via activation of the AMPK–miR-107–Eomes–PD-1 pathway (256). A metformin-containing hydrogel scaffold was developed to sustain metformin and CAR T cell release. CAR T cells reacted to metformin by markedly increased oxidative metabolism leading to a long-lived, highly activated phenotype, that enhanced anti-tumor efficacy (257). Conversely, metformin inhibited CD19 CAR T cell proliferation and cytotoxicity, while inducing apoptosis via AMPK pathway. Metformin treatment also suppressed cytotoxicity of CD19-CAR T cell *in vivo* (316). However, its exact mechanisms of action remain unclear, and the potential immunomodulatory anti-tumor effects of metformin are still under investigation.

#### 4.1.12 NAD^+^ precursors

NAD^+^, a crucial coenzyme in multiple metabolic processes, has shown health benefits, such as extended lifespan (317). Maintaining sufficient intracellular NAD^+^ is vital for cell metabolism, redox balance, and NAD^+^-dependent pathways. NAD^+^ also critically regulates NAD^+^-consuming enzymes like sirtuins (318), implicated in cellular aging. Nicotinic acid, nicotinamide mononucleotide (NMN), nicotinamide and nicotinamide riboside (NR) belong to the group of vitamins B3 and are used for NAD^+^ biosynthesis. In cancer immunotherapies, NR supplementation enhanced T cell mitochondrial fitness and improved responsiveness to anti-PD-1 treatment (296). Conversely, nicotinamide inhibited T cell exhaustion and increased differentiation into CD8^+^ effector subtypes (319). One study showed NAD^+^ depletion suppressed glycolysis, disrupted mitochondrial function, and dampened ATP synthesis. Both adoptive CAR T and anti-PD1 immune checkpoint blockade mouse models demonstrated NAD^+^ supplementation could enhance the tumor-killing efficacy. This research identified a compromised TCR-TUB-NAMPT-NAD+ pathway as a contributor to T cell dysfunction within tumors, suggesting that NAD^+^ nutrient supplements could enhance T cell-based immunotherapy (320). Although CAR T cells expressing NADH oxidase (converting NADH to NAD^+^) demonstrated increased oxygen and lactate consumption along with higher pyruvate production, they did not exhibit enhanced anti-tumor effectiveness in an *in vivo* mesothelioma model (321). However, NMN treatment significantly increased T_scm_ and T_cm_ subpopulations of CD19 CAR T cells, enhancing efficacy and longevity via the NAD^+^–Sirt1 axis (pre-print) (322). A very recent study demonstrated that aged T cells with reduced NAD cellular levels, are linked to decreased mitochondrial fitness, ultimately preventing the maintenance of stem-like properties of CAR T cells leading to deficient *in vivo* long-term survival. This research indicated that with targeting NAD^+^ pathway, it is possible to recover the mitochondrial fitness and functionality of CAR T cells. However, the sole administration of NAD precursors (NMN or NR) was insufficient in older adults. Combining strategies to increase NAD^+^ levels with a CD38 or PARP inhibitor, revitalized aged CAR T cells, thereby enhancing their therapeutic effectiveness (258).

#### 4.1.13 Modulators of lipid metabolism

Previous research has shown that membrane lipids can directly control T cell signaling and function. Cholesterol, a key component of membrane lipids, is essential for TCR clustering, immunological synapse formation and T cell proliferation (323–325). Cholesterol acyltransferase 1 (ACAT1) is the primary enzyme responsible for cholesterol esterification in CD8^+^ T cells, and blocking its activity can significantly enhance the cells’ effector function. Inhibiting ACAT1 in mesothelin CAR T cells using siRNA enhanced their efficacy against pancreatic cancer (326). Avasimibe, a repurposed drug currently under clinical development, effectively blocks ACAT1, leading to an elevated anti-tumor response from CD8^+^ T cells and superior results when combined with anti-PD1 therapy (327). Avasimibe use substantially heightened cytotoxic effect of CD19 directed CAR T cells on K562 target cells, with IFN-γ increased in nearly half the cases (260). Using Avasimibe presents a promising new approach by regulating T cell metabolism to improve anti-tumor efficacy, given the known safety profiles in humans. Another drug, Fenofibrate, is an FDA-approved peroxisome proliferator-activated receptor-α (PPAR-α) agonist, used for treating hyperlipidemia (328), cardiovascular events (329), diabetes (330), liver diseases (331) and cancer (332). Fenofibrate exerts a direct anti-inflammatory action by activating PPAR-α, which is crucial for managing lipid metabolism and inflammation (333). In addition to suppressing inflammatory pathways, Fenofibrate can preserve mitochondrial function and promote the differentiation of Tregs *in vivo* (334). In a mouse melanoma model, combining a T cell–inducing cancer vaccine with Fenofibrate improved the ability of vaccine-induced CD8^+^ TILs to delay tumor progression (335). A recent study with Fenofibrate revealed a transition from glycolysis to FAO in CD8^+^ cells, and showed that Fenofibrate-treated mice exhibited a slower progression of melanomas (336). However, the drug’s impact on CAR T cell therapy remains unclear but holds promise for further research.

#### 4.1.14 Resveratrol

With its broad potential uses, promising therapeutic impact for numerous diseases, and nutraceutical properties, resveratrol is a compound with sirtuin-activating effect. Literature reveals considerable clinical evidence demonstrating its antioxidant, anticarcinogenic, anti-inflammatory, neuroprotective, cardioprotective, and anti-aging properties (337–339). Resveratrol stimulates Sirt1 activity, which decreases c-Jun acetylation, consequently inhibiting the activation and cytokine production of T cells *in vitro* and *in vivo* (340). In addition, resveratrol has demonstrated improvement in multiple experimental autoimmunity models (341). Additionally, it counteracted colorectal cancer in a mouse model, increasing anti-inflammatory cells (CD4^+^ Tregs and CD4^+^ IL-10^+^ cells), and decreased pro-inflammatory cells such as T helper (Th)1 and Th17 cells (342). Resveratrol also enhanced anti-tumor cytokine production, while reducing tumor growth factor (TGF)-β levels. It promoted the polarization of CD4^+^ T cells and macrophages towards anti-cancer cells with diminishing the invasion and polarization of immunosuppressive cells (343). In 2021, it was demonstrated that the resveratrol-repressible CAR expression (RES_rep_-CAR) device can suppress CAR expression and CAR-driven cell activation in primary human T cells and a leukemia xenograft mouse model, with the ability to decrease T cell activation in case of severe toxicity (261).

### 4.2 Effect of nutrient levels on CAR T cell production

**Figure 4:**
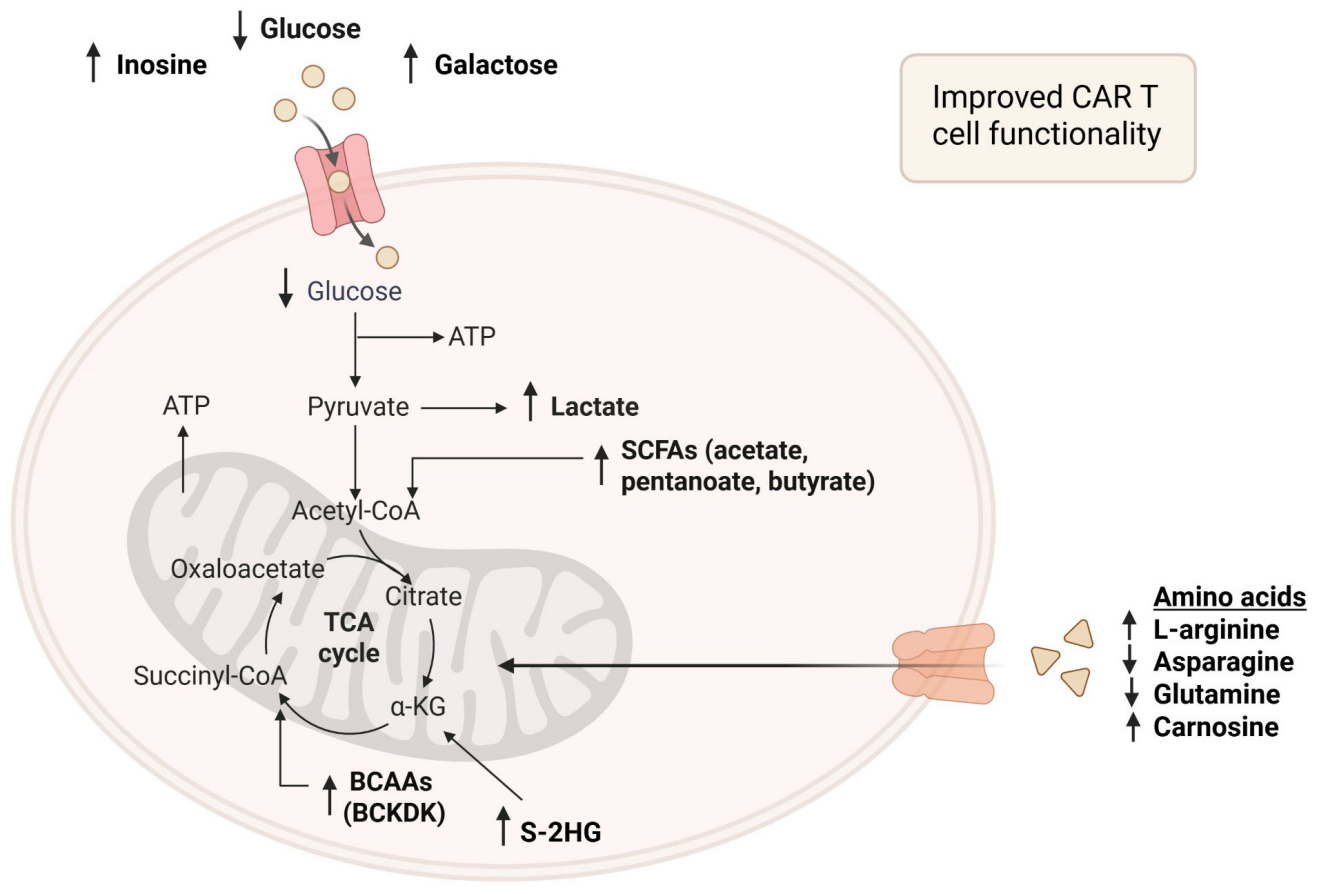
Schematic representation of boosting CAR T cell functionality with adjusting nutrient levels and metabolites within the cell. Key processes include transient restriction of glucose and the substitution of glucose for another carbohydrate such as galactose or inosine. Media supplemented with SCFAs (acetate, pentanoate and butyrate), which can be converted into acetyl-CoA, and then participate in mitochondrial oxidation, can also decrease dependence on glycolysis. Furthermore, the addition of amino acids (e.g. L-arginine, carnosine or BCAAs) or glutamine/asparagine depletion could be beneficial. Finally, the regulation of metabolites such as lactate or S-2HG could contribute to enhanced CAR T cell activity and functionality.

To improve the efficacy of T cell adoptive transfer therapy, optimizing culture medium nutritional components during manufacturing process is crucial. Specific components of media that facilitate the development of functional and persistent T cells are still under investigation. A simplified schematization of how nutrient restriction or addition could improve CAR T cell functionality is presented in **Figure 4**.

#### 4.2.1 Glucose

Glucose is essential component for T cells proliferation and effector functions. Expanding CAR T cells in glucose-restricted media augments CAR T cell function and T_cm_ phenotype (344). Limiting glucose reduces calcium and NFAT signaling, thereby impairing T cell function (345). However, recently it was shown that a period of glucose restriction after activation during CAR T expansion could be beneficial. Transient glucose restriction (TGR) in activated CD8^+^ T_eff_ cells improved their effector functions and increased tumor clearance in a lymphoma mouse model. Specifically, glucose exposure following TGR significantly increases IFN-γ, granzyme B, and CD25 expression in CD8^+^ T cells compared to T cells not undergoing TGR. The mechanism involves metabolic remodeling, shifting metabolism towards OXPHOS, with increased glucose uptake, carbon allocation to PPP, and a cellular redox shift toward a reduced state (346). Implementing TGR during the production of CAR T cells can help minimize TCR stimulation-triggered differentiation, leading to a more efficient and effective process. Furthermore, studies show enforced expression of the glucose transporter GLUT1 enhances anti-tumor efficacy and promotes favorable CAR T cell phenotypes. GLUT1 promotes T_scm_ formation and prolongs survival with decreased exhaustion markers (347). Some evidence also shows that enforced expression of high-affinity glucose transporter GLUT3 in primary murine CD8^+^ T cells can improve metabolic fitness, effector functions and survival. Moreover, a proportion of treated mice were cured and protected from re-challenge, indicative long-term T cell persistence and memory formation (348).

#### 4.2.2 Galactose

The substitution of glucose for another carbohydrate such as galactose in cell culture media has previously been demonstrated to boost OXPHOS levels in T cells and NK cells. Galactose, a carbon source, is more efficiently utilized through mitochondrial metabolism than glycolysis. It has been used to compel cells to reduce their glycolytic metabolism, which previously had a positive impact on T cell therapy (349, 350). Galactose-containing media enhanced the mitochondrial activity in CD19 CAR T cells, leading to improved *in vitro* efficacy, while maintaining a consistent memory profile without observable phenotypic changes. Furthermore, using an *in vivo* model of Nalm6 injected mice, galactose-primed CAR T cells significantly improved leukemia-free survival compared to standard glucose-cultured CAR T cells (351). In some experimental setups, galactose oxidase (an enzyme that modifies galactose) combined with neuraminidase, has further enhanced CAR T-cell killing of solid tumors (352).

#### 4.2.3 Inosine

A further consequence of glucose restricted T cells is the ability to utilize inosine. T cells exhibit metabolic plasticity during glucose restriction, incorporating inosine-metabolized carbons into central metabolic pathways to generate ATP and biosynthetic intermediates resulting in an enhanced oxidative metabolism (353). Additionally, T cells may utilize inosine and adenosine accumulated in the TME as an important carbon and energy source (354). Specifically, inosine is metabolized from adenosine through adenosine deaminase and adenosine was recognized as an immunosuppressive nucleoside that contributes to immune evasion in the TME (353). Supplementation with inosine has been shown to enhance T_eff_-cell-mediated anti-tumor properties in solid tumors of animal models that are defective in metabolizing inosine (353). Recent clinical trials also indicate that inosine supplementation, administered orally or intravenously, is safe and tolerable for treating multiple sclerosis and Parkinson’s disease (355). Klysz et al. investigated the impact of inosine on CAR T cell properties and demonstrated induced hallmark features of T cell stemness and metabolic reprogramming towards increased OXPHOS, glutaminolysis and polyamine synthesis, in addition to modulated epigenome. Inosine downregulated genes involved in purinergic signaling (CD39, CD73 and A2aR), ADORA2A and FOXP3 expression, showing distinct programming between adenosine and inosine. Similarly, overexpression (OE) of adenosine deaminase induced stemness and enhanced CAR T functionality (356).

#### 4.2.4 S-2HG and glutarate

Mammalian cells produce two chiral forms of the metabolite 2-hydroxyglutarate (2HG), R-2HG and S-2HG, which are typically kept at low levels in cells and body fluids by specific dehydrogenase enzymes converting them to α-KG (357). S-2HG increases upon hypoxia or mitochondrial dysfunction (358). Specific metabolites like S-2HG are intracellularly increased after TCR triggering, through a mechanism that involves HIF-1α. T cell activation in combination with hypoxia led to extremely high accumulation of intracellular S-2HG. While HIF-1α stabilization is required for a metabolic switch toward glycolysis and effector differentiation (91), HIF-1α is also responsible for increased concentrations of S-2HG. S-2HG can regulate T cell fate in mouse CD8^+^ T cells, inhibiting their differentiation into effector cells (359). A known S-2HG target is the TET2 demethylase, whose inhibition has correlated with the increased production of T_cm_ CAR T cells and complete tumor remission in a patient (187). Expanding CAR T cells in the presence of S-2HG yields increased proportions of T_cm_ and shows superior anti-tumor responses (181). In addition, researchers sought to determine whether molecules structurally similar to S-2HG might exert similar effects on T cell differentiation and function. They have found that glutarate, an important metabolite with significant T cell modulatory capacity, modifies proteins via glutarylation, particularly targeting pyruvate dehydrogenase E2 subunit (PDHE2). Glutarate and its cell-permeable form, diethyl glutarate (DEG), increased the population of T_cm_, with DEG boosting cytotoxicity in both human CAR T cells and mouse OT1 CD8^+^ T cells. Moreover, DEG-treated CAR T cells showed enhanced killing of HER2 ovarian cancer cells and reduced tumor growth in mice (360).

#### 4.2.5 Lactate

Lactate is a crucial metabolite derived from the glycolytic breakdown of glucose molecules and functions as a primary carbon fuel source for various cell types. Tumor cells and activated T cells both produce lactic acid at high rates, however in TME, the effect of lactate on cancer and immune cells is complex. Lactate is an often neglected carbon source that can sustain TCA anaplerosis and influence T cell function (361). CD8^+^ T cells have shown inhibited proliferation and cytotoxicity due to lactate (362, 363), but its exact role may be more complex, especially in conditions of glucose deprivation (364). One study suggests that T_regs_ can use lactate in TME to sustain their metabolism, providing them some advantage over conventional T cells (365). The role of lactate in promoting the development of a CAR Treg phenotype within the TME and its potential impact on anti-tumor responses remain unclear and require further investigation. One study showed that expanding CD8^+^ T cells under acidic conditions can lead to a stem-like phenotype with reduced glycolysis, increased FAO, and suppressed mTOR activity. Furthermore, *in vitro* CD8^+^ T cells pre-treated with lactate efficiently inhibit tumor growth upon adoptive transfer to tumor-bearing mice (366). Similarly, CD19 CAR T cells expanded under acidic conditions demonstrated improved tumor rejection when transferred to CD19-overexpressing K562 tumor-bearing NSG mice. Upon long-term *in vitro* exposure to acidic conditions, the frequency of TIM-3^+^ LAG-3^+^ infiltrating OT-I T cells and CD19 CAR T cells within the tumors was largely reduced after adoptive transfer, which is consistent with their lower *in vitro* exhaustion and improved tumor control capacity *in vivo* (367).

#### 4.2.6 Fatty acids

Fatty acids are predominantly used to fuel FAO and maintain SRC in memory CD8^+^ T cells, which rely heavily on OXPHOS. Notably, memory CD8^+^ T cells preferentially engage in a “futile cycle” of *de novo* fatty acid synthesis (FAS) coupled with lysosome-based lipid storage to sustain long-term survival and ensure the immediate availability of ATP during antigen re-challenge, rather than relying on the direct uptake of fatty acids from the environment (131).

##### 4.2.6.1 Long chain fatty acids

Exogenous free fatty acids play a fundamental role in T cell proliferation, differentiation, and function. Long chain fatty acids (LCFA) support the proliferation and differentiation of Th1 and Th17 polarized T cells during inflammatory immune responses, which is mediated, in part, by an increase in P38-MAPK signaling (368). Early studies showing a role of LCFA in memory T cell differentiation relied on nonspecific concentrations of etomoxir to inhibit CPT1A (369). However, another study found that the accumulation of LCFA in the TME was detrimental to T cells (370).

##### 4.2.6.2 Short chain fatty acids

Abundant data suggest that the activity of immune cells can be modulated by short chain fatty acids (SCFAs), including acetate, propionate, and butyrate, influencing T cell differentiation through mTOR activity, glucose metabolism and histone acetylation (371). In T cells, SCFAs can be converted into acetyl-CoA, participating in mitochondrial oxidation, while their supplementation can decrease dependence on glycolysis. Changes in the acetyl-CoA pool significantly impact histone acetylation levels and consequently gene expression levels. Consistent with this, RAR/RXR activation by octanoate could mediate the observed lipid metabolism-specific gene expression (372). SCFAs can also enhance long-term memory by upregulating FOXO1, a key transcription factor for memory formation via fatty acid metabolism and glutaminolysis to energize the TCA cycle, rather than glycolysis (371). Luu et al. showed that pentanoate and butyrate enhance the anti-tumor activity of human and murine cytotoxic T lymphocytes (CTLs) and CAR T cells that recognize receptor tyrosine kinase-like orphan receptor 1 (ROR1), through metabolic and epigenetic reprogramming. Pentanoate and butyrate enhanced mTOR activity and suppressed class I HDAC activity, which consequently increased production of effector molecules (CD25, IFN-γ, TNF-α). Pentanoate treated cells were further tested in a pancreatic tumor model and showed significantly reduced tumor volume and weight compared to non-treated cells (373). Besides pentanoate and butyrate, acetate could also have beneficial effect on CAR T cell functionality. While serving as an alternative carbon source in the nutrient-limited TME, it rescued effector functions in glucose-restricted CD8^+^ T cells (354) and enhanced IFN-γ gene transcription *in vitro* (272). Addition of acetate *ex vivo* promoted chromatin accessibility, histone acetylation and IFN-γ production in glucose-restricted T cells through acetyl-CoA synthetase (ACSS)-dependent production of acetyl-CoA, warranting further CAR T therapy exploration (354). Another study showed that increased systemic acetate concentrations are functionally integrated by CD8^+^ T cells and are required for optimal memory CD8^+^ T cell function *in vitro* and *in vivo* (374).

#### 4.2.7 Amino acids

The largest constituent of cell mass comes from amino acids in proteins. They are the building blocks of protein, sources of carbon or nitrogen for energy, anabolic substrates, and signaling molecules. T cells solely rely on exogenous sources of essential amino acids for growth. Although T cells synthesize all non-essential amino acids (NEAAs), robust proliferation and effector response requires an exogenous supply of NEAAs. A better understanding of T cells amino acid dependence and metabolic interplay could lead to novel approaches to improve T cell metabolic fitness and cancer immunotherapy.

##### 4.2.7.1 L-arginine

L-arginine is crucial for protein synthesis and is a precursor for metabolites like polyamines and nitric oxide (NO), which possess significant immunomodulatory effects (375). Fultang and colleagues proposed, for instance, the first metabolically modified CAR T cells with enzyme insertions of argininosuccinate synthase (ASS) and ornithine transcarbamylase (OCT) which would allow CAR T cells to refuel themselves in a low arginine microenvironment. This resulted in increased proliferation and sustained cytotoxic function for different *in vivo* pre-clinical tumor models (376). In activated T cells, the addition of arginine can promote OXPHOS and inhibit glycolysis, inducing a T_cm_-like phenotype with improved persistence and anti-tumor activity *in vivo* (377). This suggests that CAR T cells pre-incubated with L-arginine before adoptive transfer could enhance their metabolic fitness, persistence, and effector functions.

##### 4.2.7.2 Asparagine restriction

Interestingly, asparagine metabolism and it’s uptake have emerged as a key targets in cancer therapy (378). Asparaginase therapy was one of the first successful metabolic therapies used for treatment of acute lymphoblastic leukemia and lymphomas (379). A study on CD8^+^ T cells revealed asparagine restriction triggered cells to initiate *de novo* biosynthesis to restore their intracellular asparagine pool, with overall carbon usage/disposal, increased nucleotide production, and simultaneous activation of effector properties. Asparagine restriction markedly increased oxygen consumption and mitochondrial mass in CD8^+^ T cells with an overall decrease in glucose and glutamine consumption. This suggests that asparagine deficient media formulation can be incorporated into current process of manufacturing CAR T cells (380).

##### 4.2.7.3 Glutamine

Similarly to asparagine, reducing glutamine catabolism could promote OXPHOS and anti-tumoral activities of CD8^+^ T_eff_ cells. Nabe et al. reported that glutamine restriction during TCR‐mediated activation phase augments memory cells and enhances anti-tumor activity, due to increased proliferation and survival capacities of tumor‐specific CD8^+^ T cells (290). In 2024, a study on T cell receptor alpha constant (TRAC)-CAR T cells, which have an enriched T_scm_ population, was performed. TRAC-CAR T cells that were activated in glucose/glutamine-low medium with IL-7/IL-15, showed better persistence *in vivo* and *in vitro*, with enrichment of T_cm_ cells noted in the spleen, compared to the cells activated in standard glucose/glutamine media with IL-2 (381). Importantly, an additional study showed that the medium choice during TRAC-CAR T cell culture seems to impact their metabolism and phenotype more than the choice of cytokines (382). Recently, a study on BCMA-CAR T cells found that murine CAR T cells targeting BCMA in multiple myeloma cells were sensitive to glutamine deprivation. However, CAR T cells engineered to express glutamine transporter Asct2 showed enhanced proliferation, antigen responsiveness, IFN-γ production, and cytotoxicity under low glutamine conditions. Asct2 overexpression reprogrammed CAR T cell metabolic fitness by upregulating mTORC1 signature, modifying SLC transporter repertoire, and improving oxygen consumption and glycolytic function, enhancing CAR T cell persistence *in vivo* (360). This opens the door to exploring additional strategies that counteract glutamine deprivation in the TME, thereby empowering T cells to mount a more effective anti-tumor response.

##### 4.2.7.4 Carnosine

Carnosine, a biogenic dipeptide (β-alanine and L-histidine), improved CAR T cell transduction with enhanced lentiviral gene expression in activated T cells. By limiting extracellular acidification, carnosine enhanced the metabolic fitness of activated T cells, shifting their metabolic profile from glycolytic to an oxidative state. These results offer valuable insights into potential compounds that could be incorporated into media tailored for gene delivery and the overall effectiveness of T cell adoptive immunotherapies (383).

##### 4.2.7.5 Branched chain amino acids

Branched chain amino acids (BCAAs) are essential for protein synthesis, T cell proliferation and differentiation, and their action is through activating the mTORC1 (384). Recently, BCAA supplementation and genetic modulation of BCAA metabolism on the phenotype and function of CAR T cells was presented. BCAA supplementation was able to increase cancer cell killing and stimulated proliferation, as well as glucose uptake of CAR T cells. OE of Branched-Chain α-Ketoacid Dehydrogenase Kinase (BCKDK), a key enzyme in BCAA metabolism, significantly improved cancer cell lysis, while BCKDK KO resulted in inferior lysis potential. In an *in vivo* study, the administration of BCKDK-OE CAR T cells notably extended the lifespan of mice implanted with NALM6-GL cancer cells. This effect was linked to the differentiation of T_cm_ cells and the proportion of resident CAR T cells present in the peripheral blood circulation (385).

#### 4.2.8 Formate

Formate plays a crucial role in one-carbon (1C) metabolism, a system that produces and utilizes single carbon units for both biosynthesis and regulatory purposes. Serine is converted to glycine by the enzyme serine hydroxy-methyl transferase (SHMT1), which allows a transfer of methyl group from serine to tetrahydrofolate (THF), which is converted to formate by methylenetetrahydrofolate dehydrogenase (MTHFD1). Supplementation of 1C units with formate was demonstrated to improve functions of exhausted CD8^+^ T cells, with global changes in the transcriptional activity of metabolic pathways, effector function, and cell cycle in CD8^+^ T cells. Moreover, the combination of formate and anti-PD-1 enhanced CD8^+^ T cell-driven tumor suppression and improved survival rates in animals with the B16-OVA tumor model (386). Unfortunately, there are no studies until now, that would investigate the effect of formate in relation to CAR T therapy.

## 5 Discussion

CAR T cell therapy has transformed hematological malignancy treatment, yet its efficacy against solid tumors is limited by the immunosuppressive and metabolically hostile TME. This review highlights that CAR T cell metabolic fitness is a critical determinant of their functional compromise, leading to exhaustion and poor persistence. This underscores a paradigm shift: cellular metabolism profoundly regulates T cell fate and function, firmly positioning metabolic reprogramming as a cornerstone for optimizing CAR T cell efficacy. Our article explored how T cell differentiation is linked to metabolic states, from quiescent OXPHOS-dependent naïve cells to glycolytic effector cells and FAO-fueled memory subsets. The therapeutic objective is to modulate CAR T cell differentiation towards less exhausted, more persistent memory phenotypes (T_scm_ and T_cm_), consistently associated with superior anti-tumor responses. Metabolic shift towards OXPHOS is often accompanied by epigenetic changes, such as increased histone acetylation and altered transcription factor activity, which are tightly connected to metabolic state and need further explanation. A major barrier to combat is T cell exhaustion, a dysfunctional state marked by poor proliferation, reduced cytokine production, and upregulation of inhibitory receptors (e.g., PD-1, LAG-3), which is tightly linked to both metabolic stress and epigenetic reprogramming, locking them into a dysfunctional state.

In this review, we point out that metabolic inhibitors hold significant potential to reprogram CAR T cell metabolism *ex vivo*. Agents targeting glycolysis (e.g., 2DG, LDH inhibitors) or the PI3K/Akt/mTOR pathway (e.g., Rapamycin, PI3Kδ/γ inhibitors), consistently promote a memory-like phenotype by shifting reliance to OXPHOS. Novel strategies targeting glutamine metabolism (6-DON), mitochondrial dynamics (Mdivi-1), mitophagy (Urolithin A, spermidine), biogenesis (Bezafibrate), and pyruvate import (MPC inhibitors) further enhance CAR T cell characteristics. Manipulating enzymes like IDH2 (Enasidenib) and ACAT1 (Avasimibe) can epigenetically reprogram cells, fostering memory and exhaustion resistance. Even repurposed drugs like metformin and DCA warrant further investigation for their immunomodulatory effects.

Beyond inhibitors, nutrient level optimization during *ex vivo* expansion directly sculpts CAR T cell metabolic profiles. Strategies like transient glucose restriction or galactose substitution promote cellular reliance on OXPHOS. Supplementation with L-arginine or restricted asparagine and glutamine can improve cell’s persistence. Research also suggests inosine, short-chain fatty acids (SCFAs) and formate may further enhance the range of nutrient-based interventions that support metabolic fitness and anti-tumor activity. Finally, cytokine selection and engineering remain critical. While IL-2 drives potent effector functions, IL-7, IL-15, or IL-21 are preferred for fostering memory phenotypes and *in vivo* persistence. Engineering CAR T cells to express beneficial cytokines (e.g., IL-15, IL-12) or understanding the complex roles of others (e.g., IL-10 paradoxically enhancing OXPHOS) offers another layer of control.

In summary, integrating metabolic engineering into the CAR T cell manufacturing process, is key to generating more potent, persistent, and functionally resilient products, expanding CAR T cell therapy’s curative potential to a broader range of cancers.

## 6 Current research gap and future perspectives

While the field has made significant progress in understanding the link between T cell metabolism and function, major questions remain, particularly concerning the translation of these findings into clinical breakthroughs. A deeper understanding is needed to fully optimize CAR T cell function and persistence.

Currently, the most prominent research gaps and future perspectives to fully translate these metabolic insights into clinical breakthroughs are:

Understanding the Interplay Between Metabolism, Epigenetics, and Transcription: A deeper elucidation of the precise molecular mechanisms by which metabolic modulations influence CAR T cell fate, particularly their intricate interplay with epigenetic modifications (e.g., the role of acetyl-CoA in histone acetylation), is paramount. For instance, while it is known that metabolites like acetyl-CoA can influence histone acetylation, the full network of metabolic enzymes and their epigenetic targets is not completely mapped. A deeper understanding of how these signals converge to program T cell fate into memory *vs*. exhausted phenotype is needed to design more precise interventions.

Optimizing the Timing, Dosage, and Combination of Metabolic Modulators: Most studies have tested single metabolic inhibitors or nutrients in isolation. A major gap is the lack of knowledge regarding the optimal combinations, dosages, and temporal application of these modulators during CAR T cell manufacturing. For example, when should a glycolysis inhibitor be introduced? For how long? And what other agents should it be paired with to achieve a synergistic effect without causing off-target toxicity or functional impairment?

Translational Validation and Biomarkers: Bridging the gap from preclinical models to clinical application requires addressing several critical factors. We need to better understand how CAR T cells engineered for improved metabolism behave in a patient’s body and the precise effects of these strategies on CAR T cell functionality within the living TME. There is a pressing need for reliable biomarkers that can predict and monitor the metabolic fitness of CAR T cells in patients. This would enable the development of personalized treatment strategies and real-time adjustments to therapy.

Addressing Patient and Tumor Heterogeneity and T cells exhaustion: Current research often relies on T cells from healthy donors, but T cells from cancer patients often have pre-existing metabolic and functional defects with an exhausted profile due to prior treatments. Therefore, better *in vitro* and *in vivo* models of T cell exhaustion are needed to validate specific metabolic modulations in the most realistic setting. Furthermore, tailoring metabolic optimization strategies to an individual patient’s baseline T cell health and the specific metabolic landscape of their tumor is a key unmet need. Future development of CAR T cell therapies for solid tumors should also optimize the metabolic phenotype of the CAR T product to match the specific TME for optimal anti-tumor action.

Integration with Advanced Cell Engineering: Future endeavors will undoubtedly focus on synergistically combining metabolic engineering strategies with cutting-edge CAR designs, such as next-generation CARs, logic-gated CARs, or multi-specific approaches. This integration, along with complementary immunotherapies like checkpoint blockade or oncolytic viruses, is vital for overcoming the barriers currently limiting CAR T cell efficacy in solid tumors.

Implementation of the metabolic modulation within the clinical GMP CAR T production-protocol:

Implementing metabolic modulation strategies—such as adding specific modulators or nutrient supplements—within a clinical GMP protocol for CAR T cell manufacturing is highly feasible and supported by emerging translational research. These interventions are easily integrated because metabolic modulators and optimized nutrients can be added during the expansion phase using standard GMP-compatible media. Many modulators are already available in GMP-grade formulations, simplifying regulatory approval, and these strategies would not require major changes to existing closed-system protocols. Such alterations would likely require the addition of some metabolic functional assays and Quality Control (QC) standards, which would increase initial costs. However, these initial costs would be low relative to the overall cost of CAR T therapy. Since metabolic modulation strategies are designed to enhance cell quality, persistence, and therapeutic efficacy of T cells, this investment has the potential to dramatically reduce the need for costly subsequent interventions, ultimately leading to lower overall long-term treatment costs of CAR T therapy and improved patient benefit.
